# HiCForecast: dynamic network optical flow estimation algorithm for spatiotemporal Hi-C data forecasting

**DOI:** 10.1093/bioinformatics/btaf030

**Published:** 2025-01-22

**Authors:** Dmitry Pinchuk, H M A Mohit Chowdhury, Abhishek Pandeya, Oluwatosin Oluwadare

**Affiliations:** Department of Computer Science, University of Wisconsin-Madison, Madison, WI 53706, United States; Department of Computer Science, University of Colorado, Colorado Springs, CO 80918, United States; Department of Computer Science, University of Colorado, Colorado Springs, CO 80918, United States; Department of Computer Science, University of Colorado, Colorado Springs, CO 80918, United States; Department of Biomedical Informatics, University of Colorado, Anschutz Medical Campus, Aurora, CO 80045, United States

## Abstract

**Motivation:**

The exploration of the 3D organization of DNA within the nucleus in relation to various stages of cellular development has led to experiments generating spatiotemporal Hi-C data. However, there is limited spatiotemporal Hi-C data for many organisms, impeding the study of 3D genome dynamics. To overcome this limitation and advance our understanding of genome organization, it is crucial to develop methods for forecasting Hi-C data at future time points from existing timeseries Hi-C data.

**Result:**

In this work, we designed a novel framework named HiCForecast, adopting a dynamic voxel flow algorithm to forecast future spatiotemporal Hi-C data. We evaluated how well our method generalizes forecasting data across different species and systems, ensuring performance in homogeneous, heterogeneous, and general contexts. Using both computational and biological evaluation metrics, our results show that HiCForecast outperforms the current state-of-the-art algorithm, emerging as an efficient and powerful tool for forecasting future spatiotemporal Hi-C datasets.

**Availability and implementation:**

HiCForecast is publicly available at https://github.com/OluwadareLab/HiCForecast.

## 1 Introduction

The 3D structure of chromatin is crucial for researchers studying the relationships between chromatin architecture and gene regulation, expression, and transcription (Rao *et al.* 2014). Many biological processes in a cell are time-dependent, and analysing 3D chromatin structure as it evolves over time is crucial to understanding them. Studying the dynamic behavior of chromatin over time can provide insights into epigenetic regulation, such as why certain genes are upregulated or downregulated during embryonic development or when comparing diseased cells with healthy cells (Dixon *et al.* 2016).

3D chromatin structure is typically reconstructed using high-throughput chromatin conformation capture methods, such as Hi-C. The Hi-C method measures the frequency of contact between different loci within the genome, which are segments of DNA. This contact data is represented by an n×n Hi-C contact matrix, where *n* represents the number of loci, and the (i,j)th entry represents the contact frequency between loci *i* and *j*. These loci could be on the same or different chromosomes, providing insights into both intra- and inter-chromosomal interactions. The number of loci depends on factors such as restriction cut sites, binning, and the resolution of the data (Lieberman-Aiden *et al.* 2009).

While Hi-C experiments provide valuable snapshots of chromatin conformation at specific time points, reconstructing the time-evolving 3D structure (often referred to as 4D chromatin structure) remains a challenge. Forecasting future Hi-C data from prior time points could not only improve our understanding of temporal chromatin dynamics but also offer a faster and more cost-effective alternative to laboratory-based experimental techniques, especially for cells with limited spatiotemporal Hi-C data, to study chromatin structures at various times and stages (Liu and Wang 2023).

Several studies have explored the interpolation of 3D chromosome structures between two given time points including TADdyn (Di Stefano *et al.* 2020) and 4DMax (Highsmith and Cheng 2021). However, currently there is only one method named HiC4D (Liu and Wang 2023) that focuses on forecasting future Hi-C data based on Hi-C timeseries. The HiC4D method treats Hi-C contact matrices as frames of a video and employs a long short-term memory (LSTM) based video prediction algorithm. Specifically, Liu and Wang developed the ResConvLSTM model by adding residual skip connections between ConvLSTM (Shi *et al.* 2015) layers.

In this work, we propose to create a robust and dynamic algorithm based on contextual understanding of the previous steps using optical flow for spatiotemporal Hi-C forecasting. The utilization of optical flow distinguishes our approach from others, as it serves a critical role in our algorithm, setting it apart from existing methods. Specifically, we propose a more robust framework called HiCForecast where we predict the expected optical flow in future timeseries Hi-C data through a dynamic and context based prediction model using a dynamic voxel flow network model (Hu *et al.* 2023). HiCForecast allows us to model complex relationships in timeseries transitions between loci through dynamic optical flow estimation.

## 2 Materials and methods

### 2.1 Architecture

We interpret the Hi-C timepoints as frames of a video in order to apply a video prediction algorithm to them (Liu and Wang 2023). HiCForecast adapts the dynamic multi-scale voxel flow network (DMVFN) (Hu *et al.* 2023) video prediction algorithm to predict future Hi-C contact data from a series of existing time-frames. It takes two frames as input and predicts the next three. The model consists of MVFB blocks that use estimated optical flow to predict the next frame. An MVFB block takes the output of a previous MVFB block together with two input frames to synthesize the next frame by using optical flow estimation. The optical flow and predicted next frame are continuously refined as they pass through the chain of MVFB blocks until they turn into the final prediction.

#### 2.1.1 Optical flow setup

Optical flow is the per pixel motion between two frames of a video, which can be used to reconstruct the next frame given the previous one. Specifically, suppose the input frames are $I_{t-1}$ and $I_{t}$ and the goal is to predict frame $I_{t+1}$. DMVFN estimates the optical flows $f_{t+1\to t}$ and $f_{t+1\to t-1}$ from $I_{t+1}$ to the two input frames. Using the backward warping operation $\overset{\leftarrow }{W}$ we can make the following estimates

(1) $${\hat{I}}_{t+1\leftarrow t-1}=\overset{\leftarrow }{W}(I_{t-1},f_{t+1\to t-1})$$

 

(2) $${\hat{I}}_{t+1\leftarrow t}=\overset{\leftarrow }{W}(I_{t},f_{t+1\to t})$$

A binary mask *m*, which is estimated by the algorithm, is then used to combine these two estimates into a single prediction as follows:

(3) $${\tilde{I}}_{t+1}=m\times {\hat{I}}_{t+1\leftarrow t-1}+(1-m)\times {\hat{I}}_{t+1\leftarrow t}$$

Denoting $F_{t+1}=(f_{t+1\to t-1},f_{t+1\to t},m)$ we can collectively denote Equations (1)–(3) as

(4) $${\tilde{I}}_{t+1}=\overset{\leftarrow }{W}(I_{t-1},I_{t},F_{t+1})$$

#### 2.1.2 Dynamic multi-scale voxel flow network

**Figure 1.**
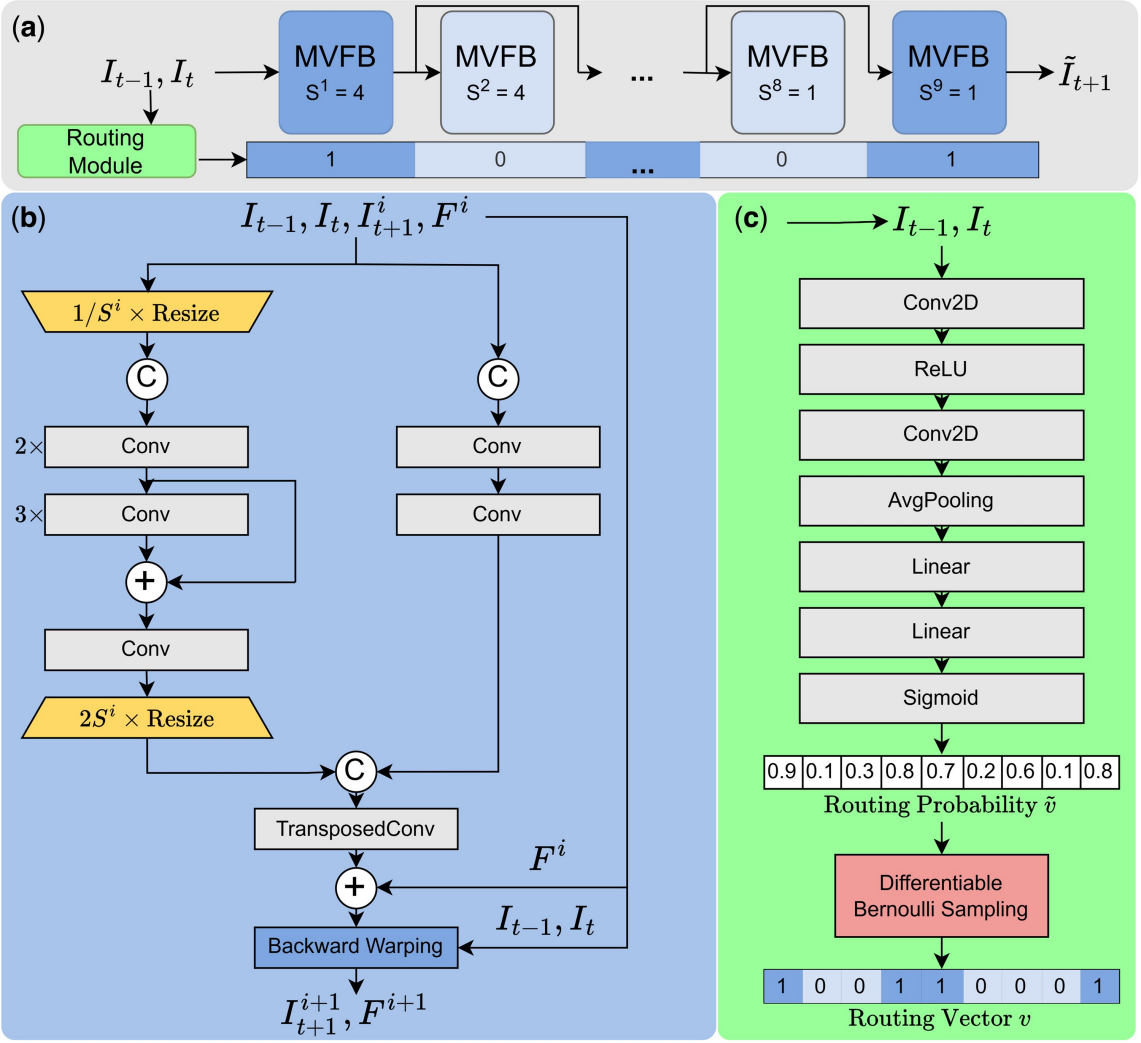
HiCForecast architecture. The model reconstructs the next frame given the input by predicting the optical flow to the next frame. (a) Overview: MVFB blocks, dynamically selected by the routing module during inference, continuously refine the optical flow to predict the next frame. (b) MVFB: each block refines the optical flow and the next frame prediction using the original input and previous optical flow, utilizing scaled and unscaled data through convolutional layers. (c) Routing module: it selects which MVFB blocks to include in the architecture at inference by generating a routing vector using input frames processed through convolutional and fully connected layers with differentiable Bernoulli sampling.

HiCForecast adapted the main architectural flow from the DMVFN (Hu *et al.* 2023) and retained most of its features through the fine tuning process in hyperparameter search. The model has a chain of MVFB blocks (Fig. 1a) each of which scales the input by some factor. A routing module adaptively selects which of these blocks will be included in the model for a particular input (Fig. 1c). The final estimate of the optical flow from the MVFB blocks together with the first two images is used to reconstruct the next frame. Let ${\tilde{I}}_{t+1}^{i-1}$ be the predicted frame from the block i−1. The *i*th MVFB block takes $I_{t-1},I_{t},{\tilde{I}}_{t+1}^{i-1},F^{i-1}$, and a scaling factor $S^{i}$ to predict ${\tilde{I}}_{t+1}^{i}$ and $F^{i}$.

Each block has a motion and a spatial path through which $F^{i-1}$ and ${\tilde{I}}_{t+1}^{i-1}$ are concatenated and passed through. The motion path resizes the input by $1/S^{i}$ and then applies six convolutional layers each equipped with *Parametric ReLU (PReLU)* activations. The output is then resized by $2S^{i}$. The outputs of the spatial and motion paths are then concatenated, and a transposed convolution is used to estimate $f_{t+1\to t-1}^{i}$ and $f_{t+1\to t}^{i}$. Then backward warping (Equation (4)) is applied to $I_{t},I_{t-1}$ with the use of this estimated optical flow to make the prediction ${\tilde{I}}_{t+1}^{i}$ (Fig. 1b).

The routing module (Fig. 1c) takes $I_{t}$ and $I_{t-1}$ as input to dynamically select which MVFB blocks will be used during inference. The inputs are concatenated and then passed through a convolution, an average pooling, and linear layer followed by a sigmoid. Bernoulli sampling is then applied to these probabilities to create the routing vector of 0’s and 1’s indicating which MVFB block will be turned on or off during inference. In order for the Bernoulli sampling to be differentiable for training purposes we use the straight-throughput estimation of Bernoulli sampling (STEBS) technique employed by DMVFN. The forward pass of the differentiable Bernoulli sampling is computed as such:

(5) $$\tilde{w_{i}}=\min\{\frac{1}{2}n\sigma (\tilde{v_{i}})/\sum_{i=1}^{n}\sigma (\tilde{v_{i}}),1\}$$

 

(6) $$v_{i}∼\text{Bernoulli}(\tilde{w_{i}})$$

where the routing probability vector produced by the sigmoid layer is denoted as $\tilde{v}$, n=9 is the number of MVFB blocks, σ is the sigmoid function, and *v* is the resulting routing vector. During the backward pass, the derivatives with respect to $\tilde{w}$ are estimated by the well-defined derivatives with respect to *v*:

(7) $$\frac{\partial L}{\partial \tilde{w}}=\frac{\partial L}{\partial v}$$

where *L* is denoting the loss function.

### 2.2 Loss function

We have used different loss functions in our hyperparameter search (Supplementary Table S1). In general, the loss function works with the predictions for the next frame, ${\tilde{I}}_{t+1}^{i}$, produced by the *i*th MVFB block. The loss functions used has the following general framework:

(8) $$L=\sum_{i=1}^{n}0.8^{n-i}d({\tilde{I}}_{t+1}^{i},I_{t+1})+\alpha L_{\text{VGG}}({\tilde{I}}_{t+1},I_{t+1})$$

where *n* is the number of MVFB blocks, $L_{\text{VGG}}$ is the VGG loss (Ledig *et al.* 2017), *d* was taken to be either the $l_{1}$ loss, MSE loss or the $l_{1}$ loss on the Laplacian pyramid representations (Paris *et al.* 2011) as is default in DMVFN, and the parameter α for including or excluding $L_{\text{VGG}}$ is either 0 or 0.5. For i<9 the input to the *i*th block which computes ${\tilde{I}}_{t+1}^{i}$ is computed with a chain of MVFB blocks selected by the routing module, and the *i*th block is included regardless of its presence in the routing vector. The last output ${\tilde{I}}_{t+1}^{9}$ is the result of a regular forward pass including MVFB blocks according to the routing module.

### 2.3 Data

We used the following spatiotemporal Hi-C datasets of mouse, human, medaka, and Xenopus tropicalis cells undergoing embryogenesis and cell reprogramming. We directly used the preprocessed datasets in the study from HiC4D (Liu and Wang 2023).

1. Dataset 1 (Du *et al.* 2017) is Mouse Preimplantation Embryogenesis. The time points correspond to the pronuclear stage 5 (PN5) zygote, early 2-cell, late 2-cell, 8-cell, inner cell masses (ICMs), and mouse embryonic stem cells (mESC) stages.
2. Dataset 2 (Ke *et al.* 2017) is Mouse Embryogenesis. We used the zygote, 2-cell, 4-cell, 8-cell, embryonic day (E)3.5, and E7.5 stages in our study.
3. Dataset 3 (Chen *et al.* 2019) is Human Embryogenesis. We utilized the six timesteps in the Hi-C timeseries corresponding to the 2-cell, 8-cell, morula, blastocysts, six-week-old embryo stages, and human embryonic stem cells (hESC) stages.
4. Dataset 4 (Stadhouders *et al.* 2018) is Mouse Cell Reprogramming. It contains Hi-C data from mouse somatic cells undergoing reprogramming into pluripotent stem cells. We utilized the reprogramming stages B, Bα, D2, D4, D6, and D8 as the timesteps from this dataset.
5. Dataset 5 (Nakamura *et al.* 2021) is medaka cells before, during, and after gastrulation. We utilized six stages of medaka development: stage 11 (late blastula [2–4 k cells], 8 hours post fertilization [hpf]), stage 12 (pre-early gastrula, 10 hpf), stage 13 (early gastrula, 12 hpf), stage 14 (pre-mid-gastrula, 14 hpf), stage 18 (late neurula, 24 hpf), and stage 27 (24-somite, 54 hpf), out of the 12 stages available, as the timesteps for our Hi-C timeseries.
6. Dataset 6 (Niu *et al.* 2021) is Xenopus Tropicalis Embryogenesis. Out of the nine available stages, we selected the six TAD development stages: stage 8 (S8), stage 9 (S9), stage 10 (S10), stage 12 (S12), stage 15 (S15), and stage 23 (S23), to include in our Hi-C timeseries.

We trained our model with Mouse Embryogenesis (Du *et al.* 2017) (Dataset 1) using all chromosomes except 2, 6, and 19. We used chromosome 19 to validate our model and chromosomes 2 and 6 from all datasets to test our trained model. We selected chromosomes 2, 6, and 19 to represent a range of chromosome sizes: chromosome 2 is one of the larger chromosomes, chromosome 6 is of average size, and chromosome 19 is one of the smallest. This selection was made to ensure a generalizable validation across different chromosome sizes. In short, our algorithm was trained on Dataset 1, and the generalization was then applied to Datasets 2 through 6 to create a robust training dataset providing a diverse range of chromosomal data. This approach follows a similar methodology to an existing algorithm like HiC4D (Liu and Wang 2023), where a similar selection of chromosomes is used for training and validation, hence, ensuring that our results are robust and comparable to this study. As part of the pipeline, we cut off the data values at a certain maximum value, determined through hyperparamter search, and then normalized the data to the range [0,1] (Supplementary Table S1, normalization column).

### 2.4 Evaluation metrics

In this study, we primarily used GenomeDISCO, Pearson correlation coefficient (PCC), structural similarity index measure (SSIM), and peak signal-to-noise ratio (PSNR) evaluation metrics. Each metric was selected to provide complementary insights into the quality and biological relevance of the Hi-C predictions, with GenomeDISCO and SSIM being the most biologically relevant, while PCC and PSNR provide additional accuracy measures.

#### 2.4.1 GenomeDISCO

The main evaluation metric used in this study is GenomeDISCO (Ursu *et al.* 2018), a biological reproducibility metric. GenomeDISCO computes a concordance score between −1 and 1 indicating biological similarity between two contact maps. This method smooths the contact maps using a graph representation and calculates the similarity score on the smoothed matrices, with the random walk step parameter set to the default t=3. GenomeDISCO effectively captures biological differences by evaluating structural similarity in Hi-C contact maps, which is crucial for understanding chromatin organization. By assessing structural integrity, it accounts for the most biologically relevant features, providing a more meaningful evaluation of the Hi-C map predictions. Since HiCForecast predictions are only made for 64×64 patches along the diagonal, we averaged the GenomeDISCO score for non-zero patches along the diagonal to provide a comprehensive measure of the similarity.

#### 2.4.2 Pearson correlation coefficient

We compute the average PCC between each of the non-negative n×n patches along the diagonal, where the PCC is a coefficient *r* between −1 and 1. PCC computes score between patch *X* and patch *Y* using Equation (9)
 

(9) $$r=\frac{\sum \left(x_{i}-\bar{x_{i}}\right)(y-\bar{y_{i}})}{\sqrt{\sum {\left(x_{i}-\bar{x_{i}}\right)}^{2}\sum {\left(y-\bar{y_{i}}\right)}^{2}}}$$

where $x_{i}$ are the elements of *X*, $\bar{x_{i}}$ is the average of the values in *X*, and $y_{i}$ and $\bar{y_{i}}$ are the respective values in patch *Y*. PCC provides a measure of the linear relationship between two patches, quantifying how closely predicted Hi-C maps match the ground truth. While it primarily assesses global consistency, PCC provides valuable insight into model accuracy and its ability to capture biological patterns.

#### 2.4.3 Peak signal-to-noise ratio

We compute the average PSNR over the diagonal patches of the compared matrices. PSNR between two patches is computed using Equation (10)
 

(10) $$\text{PSNR}=10{\;\text{log}\;}_{10}\left(\frac{M^{2}}{\text{MSE}}\right)$$

where *M* is the maximum value for the image and MSE is the mean squared error of the two patches. PSNR measures the quality of predicted Hi-C maps by quantifying the difference between predicted and ground truth patches in terms of pixel-wise accuracy. It evaluates the fidelity of the reconstruction, with higher PSNR values indicating less distortion and better similarity between the predicted and ground truth matrices.

#### 2.4.4 Structural similarity index measure

We compute the SSIM between a predicted matrix *x* and the ground truth *y* using Equation (11)
 

(11) $$\text{SSIM}=\frac{\left(2\mu_{x}\mu_{y}+c_{1})(2\sigma_{xy}+c_{2}\right)}{\left(\mu_{x}^{2}+\mu_{y}^{2}+c_{1})(\sigma_{x}^{2}+\sigma_{y}^{2}+c_{2}\right)}$$

where $\mu_{x},\mu_{y}$ are the respective sample mean, $\sigma_{x}^{2},\sigma_{y}^{2}$ are the respective variance, $\sigma_{xy}$ is the covariance, $c_{1}={\left(k_{1}L\right)}^{2}$ and $c_{2}={\left(k_{2}L\right)}^{2}$ in which *L* is the dynamic range, and $k_{1}$ and $k_{2}$ are constants (Wang *et al.* 2004). SSIM evaluates the structural similarity between predicted and ground truth matrices, considering luminance, contrast, and structure (Peng *et al.* 2020), unlike PSNR, which focuses on intensity differences. This makes SSIM particularly useful for assessing how well Hi-C contact maps preserve biologically significant patterns like TADs and chromatin loops, providing valuable insights into model predictions in biological contexts.

## 3 Results

We primarily evaluated our method with the GenomeDISCO biological reproducibility metric. In addition to this metric, we measured HiCForecast correctness with PCC (Equation (9)), SSIM (Equation (11)), and PSNR (Equation (10)) evaluation metrics. The evaluation involves comparing the algorithm’s output with the ground truth across all metrics at a resolution of 40 kb. For HiCForecast we provide benchmarking for all chromosomes in Datasets 1–6 for patches of 60×60 submatrices (2.4 Mb) along the diagonal. Although HiCForecast makes predictions with patches of size 64×64, we evaluated 60×60 patches to avoid non-overlapping gaps that would lack prediction values (Supplementary Table S1). On the other hand, we evaluated HiC4D using the maximum window size allowed by their algorithm while also avoiding non-overlapping gaps, which turns out to be patches of 48×48. Table 1 shows performance across Datasets 1–4 for chromosomes 2 and 6, while Supplementary Table S2 presents results for Datasets 5 and 6. Evaluation results for HiCForecast on Datasets 2–6 across all chromosomes are available in Supplementary File S1. Table 2 contains a mapping of the artificial time points ($t_{1}$ – $t_{6}$) to their corresponding biological stage described for each dataset in Section 2.3.

**Table 1.** Test results of HiCForecast and HiC4D for predicting timesteps 4, 5, and 6 on chromosomes 2 and 6 of Datasets 1–4 evaluated with GenomeDISCO, PCC, and PSNR metrics.

			$t_{4}$		$t_{5}$		$t_{6}$	
			HiCForecast	HiC4D	HiCForecast	HiC4D	HiCForecast	HiC4D
Mouse Preimplantation Embryogenesis—Dataset 1 (Du *et al.* 2017)	chr 2	GenomeDISCO	**0.892**	0.818	**0.870**	0.835	**0.855**	0.840
		PCC	**0.969**	0.881	**0.942**	0.915	**0.937**	0.903
		PSNR	**34.931**	27.253	**36.198**	32.964	**35.135**	32.057
	chr 6	GenomeDISCO	**0.897**	0.834	**0.880**	0.861	**0.859**	0.856
		PCC	**0.968**	0.885	**0.941**	0.919	**0.931**	0.901
		PSNR	**33.534**	26.293	**34.676**	32.018	**32.932**	30.511
Mouse Embryogenesis—Dataset 2 (Ke *et al.* 2017)	chr 2	GenomeDISCO	**0.875**	0.830	**0.846**	0.845	**0.820**	0.808
		PCC	**0.965**	0.941	0.942	**0.943**	0.923	**0.926**
		PSNR	**33.701**	30.393	33.128	**33.290**	**28.719**	26.245
	chr 6	GenomeDISCO	**0.891**	0.857	0.867	**0.879**	0.832	**0.838**
		PCC	**0.964**	0.941	**0.942**	**0.942**	0.921	**0.926**
		PSNR	**32.743**	29.547	32.279	**32.292**	**27.650**	25.491
Human Embryogenesis—Dataset 3 (Chen *et al.* 2019)	chr 2	GenomeDISCO	**0.799**	0.707	**0.811**	0.769	**0.643**	0.604
		PCC	**0.819**	0.808	**0.702**	0.592	**0.712**	0.359
		PSNR	**31.864**	28.676	**32.898**	30.686	**28.652**	27.032
	chr 6	GenomeDISCO	**0.795**	0.717	**0.829**	0.784	**0.658**	0.621
		PCC	**0.819**	0.808	**0.704**	0.583	**0.714**	0.347
		PSNR	**29.198**	25.986	**30.851**	28.514	**25.744**	24.094
Mouse Cell Reprogramming—Dataset 4 (Stadhouders *et al.* 2018)	chr 2	GenomeDISCO	**0.855**	0.807	**0.816**	0.738	**0.840**	0.801
		PCC	**0.968**	0.898	**0.956**	0.908	**0.937**	0.902
		PSNR	**30.426**	26.581	**26.877**	23.534	**28.492**	25.972
	chr 6	GenomeDISCO	**0.870**	0.851	**0.833**	0.779	**0.853**	0.822
		PCC	**0.969**	0.909	**0.958**	0.915	**0.939**	0.905
		PSNR	**30.473**	26.781	**26.531**	23.305	**27.755**	25.184

The results on Datasets 2–4 are blind test generalizations to different species and systems. HiC4D takes timesteps $t_{1}$, $t_{2}$, and $t_{3}$ as input, and HiCForecast takes $t_{2}$ and $t_{3}$ as input in order for the results to be comparable. HiC4D predictions for Dataset 3 were adjusted by setting negative values to 0. Results for Datasets 5 and 6 are in Supplementary Table S2. Bold highlights indicate higher scores for the given predicted time points.

**Table 2.** The correspondence between the artificial timepoints $t_{1},\ldots ,t_{6}$ and the biological stages in Datasets 1–6 used in this study.

	$t_{1}$	$t_{2}$	$t_{3}$	$t_{4}$	$t_{5}$	$t_{6}$
Dataset 1	PN5 Zygote	Early 2-cell	Late 2-cell	8-cell	ICM	mESC
Dataset 2	Zygote	2-cell	4-cell	8-cell	E3.5	E7.5
Dataset 3	2-cell	8-cell	Morula	Blastocyst	6-week	hESC
Dataset 4	B	Ba	D2	D4	D6	D8
Dataset 5	ST11	ST12	ST13	ST14	ST18	ST27
Dataset 6	ST8	ST9	ST10	ST12	ST15	ST23

### 3.1 Hyperparameter search and training

**Figure 2.**
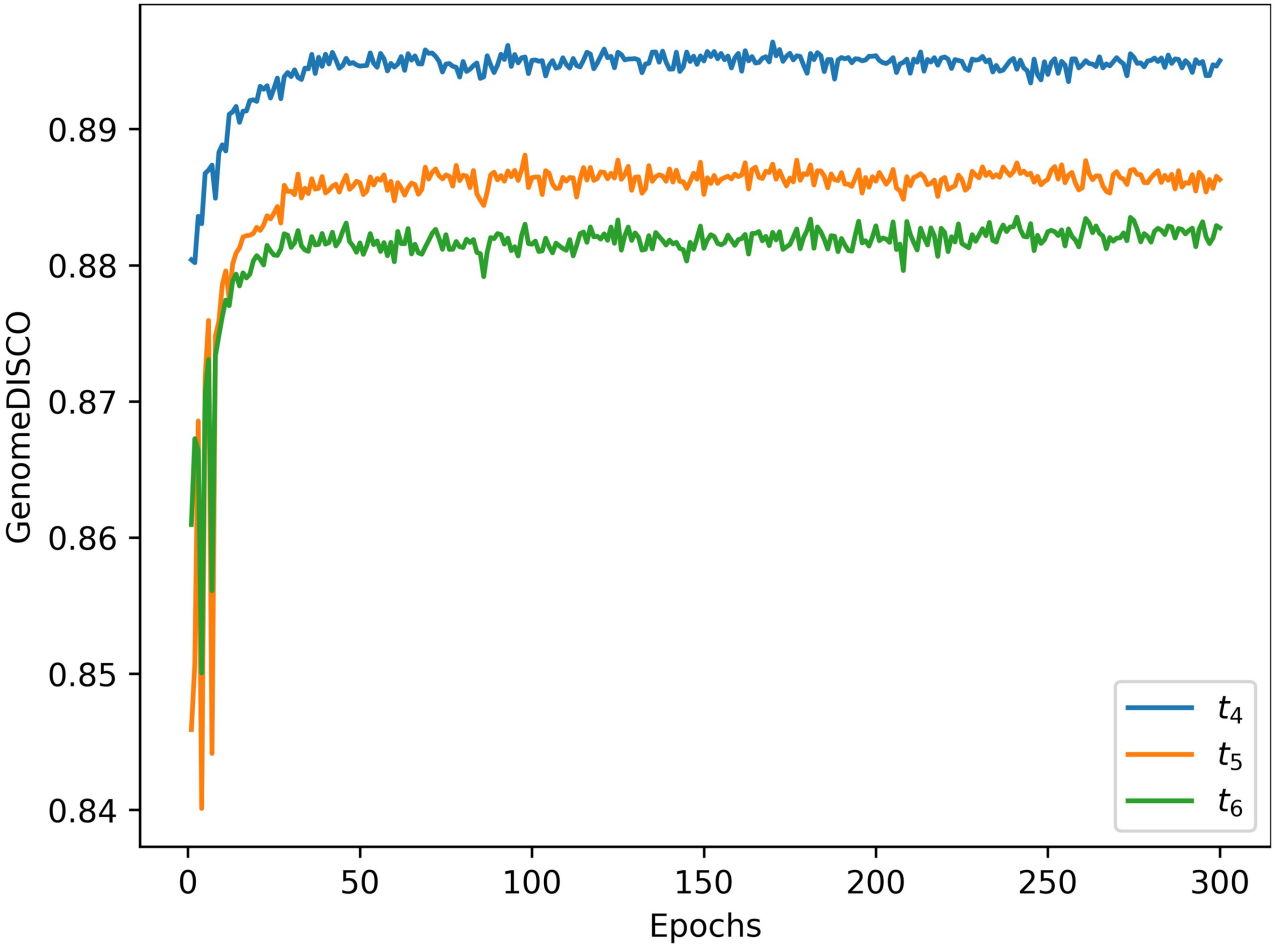
Validation GenomeDISCO scores of HiCForecast predictions for timepoints 4, 5, and 6 on chromosome 19 of Mouse Preimplantation Embryogenesis (Dataset 1) for 300 epochs of training.

We evaluated various hyperparameters, including loss functions (Equation (8)), number of MVFB blocks, the presence of routing module, window size, and normalization values (see Supplementary Table S1). Optimal performance was achieved with nine MVFB blocks and a 64×64 window size. HiCForecast was trained with a batch size of 8, using the Adam optimizer with an initial learning rate of $10^{-4}$, gradually reduced to $10^{-5}$ via cosine annealing. Model selection prioritized validation performance on timesteps $t_{4}$, $t_{5}$, and $t_{6}$, with preference given to later timesteps. Validation results for chromosome 19 of Mouse Preimplantation Embryogenesis (Dataset 1) over 300 epochs (Fig. 2) indicated a plateauing curve. For efficiency, the HiCForecast model used throughout the study was from the 100th epoch of training. Detailed hyperparameter configurations and supplementary figures are provided in Supplementary Table S1 and Supplementary Figs S1–S3.

### 3.2 Performance on the same system

#### 3.2.1 Mouse preimplantation embryogenesis (dataset 1)

**Figure 3.**
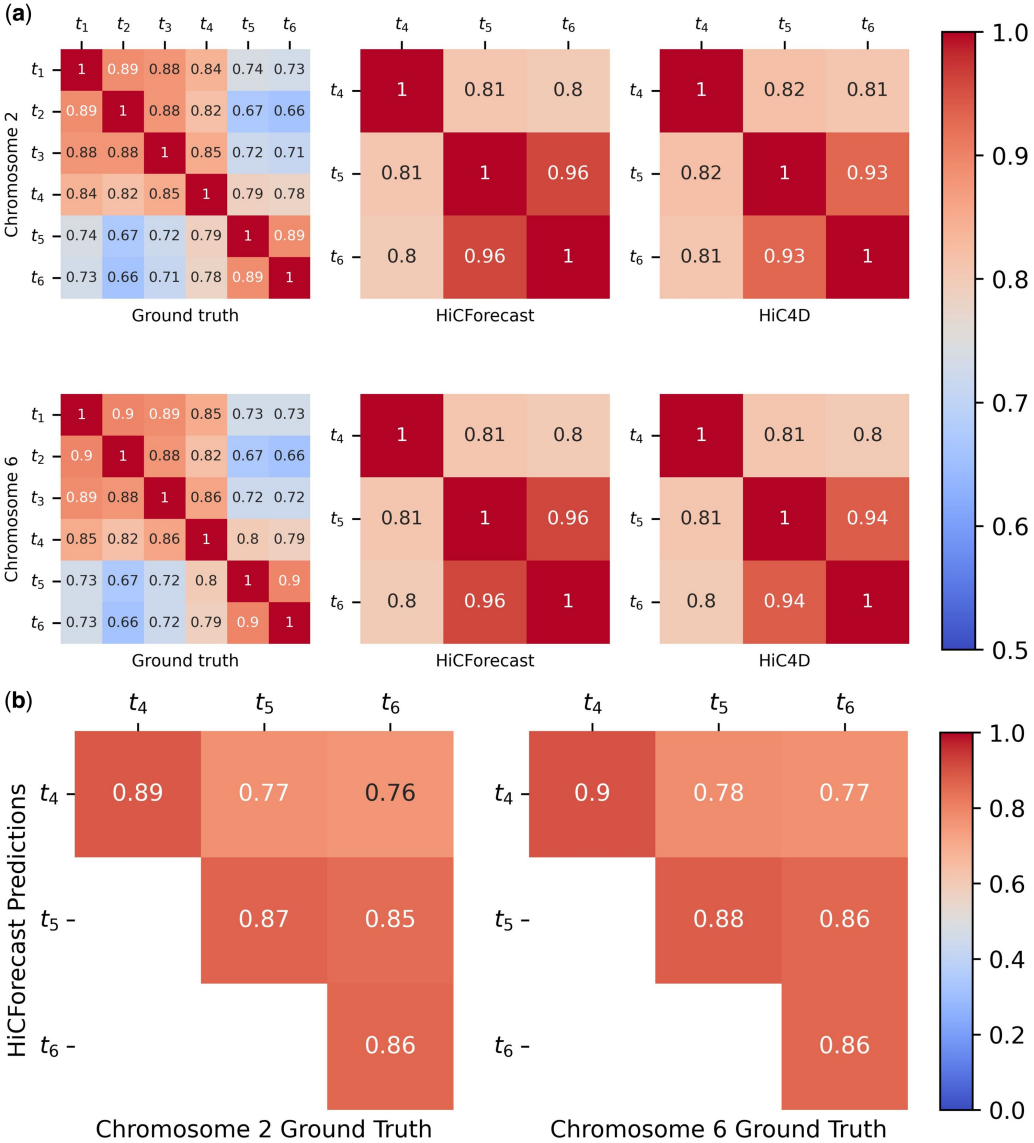
(a) GenomeDISCO scores between ground truth timesteps 1–6 and the predictions of HiCForecast and HiC4D on Mouse Preimplantation Embryogenesis (Dataset 1) chromosomes 2 and 6 demonstrating the similarity between different timesteps for both ground truth and predicted Hi-C timeseries. (b) The rows represent the timestep that HiCForecast predicted, while the column represents the ground truth timestep with which the prediction was compared with. The predictions were made for chromosomes 2 and 6 of Dataset 1, and were analysed in patches of 60 × 60 along the diagonal of the matrices.

Our blind test on this dataset demonstrates the effectiveness of our method in predicting the last three stages of embryogenesis more effectively than HiC4D, with a GenomeDISCO score greater than 0.85 across the three timesteps (Table 1). In all the future timesteps, corresponding to 8-cell, inner cell masses, and stem cell stages, HiCForecast significantly outperforms HiC4D in the remaining benchmarks, i.e. PSNR and PCC. We observed considerably higher PSNR values, suggesting that the quality of the reconstructed frames closely matches that of the future timestep signals. Next, we examined the similarity levels of Hi-C contact matrices across different timesteps (Fig. 3a).

In Fig. 3a, the first column represents the calculated GenomeDISCO scores between each pair of the six timesteps $t_{1}$ through $t_{6}$ of the ground truth data. The second column shows the pairwise GenomeDISCO similarity score between timesteps $t_{4}$ and $t_{6}$ predicted by HiCForecast, and the third column shows the same for HiC4D. Here, the timepoints $t_{1}$ through $t_{6}$ represent the PN5 zygote, early 2-cell, late 2-cell, 8-cell, ICM, and mESC stages, respectively. The goal of this figure is to demonstrate that, as observed in the ground truth (column 1), the similarity between consecutive timesteps (e.g. $t_{i}$ and $t_{i+1}$) is higher than the similarity between nonconsecutive timesteps (e.g. $t_{i}$ and $t_{i+j}$, where j>1). Hence, $t_{4}$ (8-cell stage) is similar to $t_{5}$ (ICM) than it is to $t_{6}$ (mESC). This can be observed for the ground truth for chromosomes 2 and 6. The same phenomenon is also consistently observed in predictions made by HiCForecast, highlighting the reliability of our model in preserving temporal consistency.

Additionally, we conducted a detailed analysis comparing predicted future contact matrices with ground truth data. For instance, in Dataset 1, the predicted future contact matrix for $t_{4}$ was compared against ground truth matrices of various future time steps. As shown in Fig. 3b, the predicted $t_{4}$ matrix was most similar to the ground truth $t_{4}$ matrix, rather than to matrices from more distant time steps. Similar results were observed across other predicted future time steps, as reported in the figure. These findings demonstrate that the model excels at predicting immediate future time steps, reinforcing the strength of the voxel flow approach for accurate short-term predictions.

#### 3.2.2 Mouse embryogenesis (dataset 2)

For this dataset, our test shows greater similarity to Mouse Preimplantation Embryogenesis (Dataset 1) due to factors like the number of read pairs after downsampling and their respective time-point ranking order, both from mouse embryogenesis (Table 1). Despite training on Dataset 1, our algorithm’s performance closely mirrors it, achieving high GenomeDISCO biological reproducibility scores, PCC, and PSNR values for the test chromosomes. HiCForecast is stronger than HiC4D on the first predicted timestep $t_{4}$ (8-cell stage) in all evaluation metrics; however, the results are mixed for the last two timesteps $t_{5}$ and $t_{6}$ (E3.5 and E7.5 stages) detailed in Table 1. We compared different timesteps within the predicted timeseries for this dataset similar to that in Fig. 3a (Supplementary Fig. S4).

### 3.3 Generalization to different species

#### 3.3.1 Human embryogenesis (dataset 3)

This dataset has the same system of embryogenesis as the training dataset but a different species. The GenomeDISCO performance of HiCForecast is consistently higher than HiC4D across the three predicted timesteps with a stronger performance in the first two timesteps (blastocyst and 6-week stages) (Table 1). HiCForecast is also consistently strong compared with HiC4D across all stages in PCC and PSNR. Additionally, the similarity comparison between different timesteps within the predicted timeseries for this dataset similar to those in Fig. 3a are available in Supplementary Fig. S5. The generalization across different species was also performed on Medaka Gastrulation (Dataset 5) and Xenopus Tropicalis Embryogenesis (Dataset 6) (see Supplementary Table S2). A description of their results is provided in the Supplementary Document Result section.

### 3.4 Generalization to non-embryogenesis systems

#### 3.4.1 Mouse cell reprogramming (dataset 4)

In addition to embryogenesis datasets, we also evaluated our method on datasets unrelated to embryogenesis. Despite the difference in developmental contexts, our findings reveal that spatiotemporal Hi-C data from non-embryogenesis contexts still showed impressive performance for our algorithm. HiCForecast is consistently stronger than HiC4D across all predicted stages in the GenomeDISCO, PCC, and PSNR metrics (Table 1). Even on a dataset related to mouse reprogramming, we achieved benchmark scores similar to those obtained for mouse, human, and X. tropicalis embryogenesis in datasets 2, 3, and 6. This indicates that generalization across different systems of the same species is not necessarily more difficult or easier than generalization across different species with the same dataset. This raises questions about the influence of species differences on generalization compared to differences in cell types, warranting further investigation to provide answers in future research. In addition, the comparison between different timesteps within the predicted timeseries of this dataset similar to those in Fig. 3a is available in Supplementary Fig. S6.

### 3.5 HiCForecast shows a stronger structural similarity to ground truth

To account for the importance of chromosome structure in biological function, we used the SSIM analysis to evaluate the consistency and closeness of algorithm predictions to the ground truth structure across different development stages. As discussed in Section 2.4.4, the SSIM index evaluates the preservation of biologically significant features like TADs and chromatin loops in Hi-C contact maps, making it essential for assessing model accuracy. It measures structural similarity between predicted and ground truth maps, ensuring the model captures key genomic structures. HiCForecast achieves higher index scores on average across the datasets, with some exceptions observed at specific stages, such as the blastocyst stage ($t_{4}$) on Chromosome 2 and the six-week ($t_{5}$) and hESC ($t_{6}$) stages on Chromosome 6 in Dataset 3 (Human Embryogenesis). As shown in Table 3 and Supplementary Table S4, which include four datasets, HiCForecast’s SSIM results are generally close to the ground truth, with values approaching 1. This indicates that HiCForecast predictions closely resemble the true chromatin structure and preserve more structural information compared to HiC4D.

**Table 3.** SSIM index scores for Mouse Preimplantation Embryogenesis (Dataset 1) at 40 kb resolution.

Chromosome	Timestep	HiCForecast	HiC4D
2	8-cell ($t_{4}$)	**0.9791**	0.8927
	ICM ($t_{5}$)	**0.9665**	0.8645
	mESC ($t_{6}$)	**0.9703**	0.8912
6	8-cell ($t_{4}$)	**0.9748**	0.8617
	ICM ($t_{5}$)	**0.9593**	0.8235
	mESC ($t_{6}$)	**0.9613**	0.8608

SSIM index in the range 0–1, where high is better, compares the structural similarity between predicted and ground truth maps by measuring structural preservation. HiCForecast achieved the highest score across the timesteps. The highest scores are in bold text.

### 3.6 HiCForecast identifies significant interaction in loci analysis

**Figure 4.**
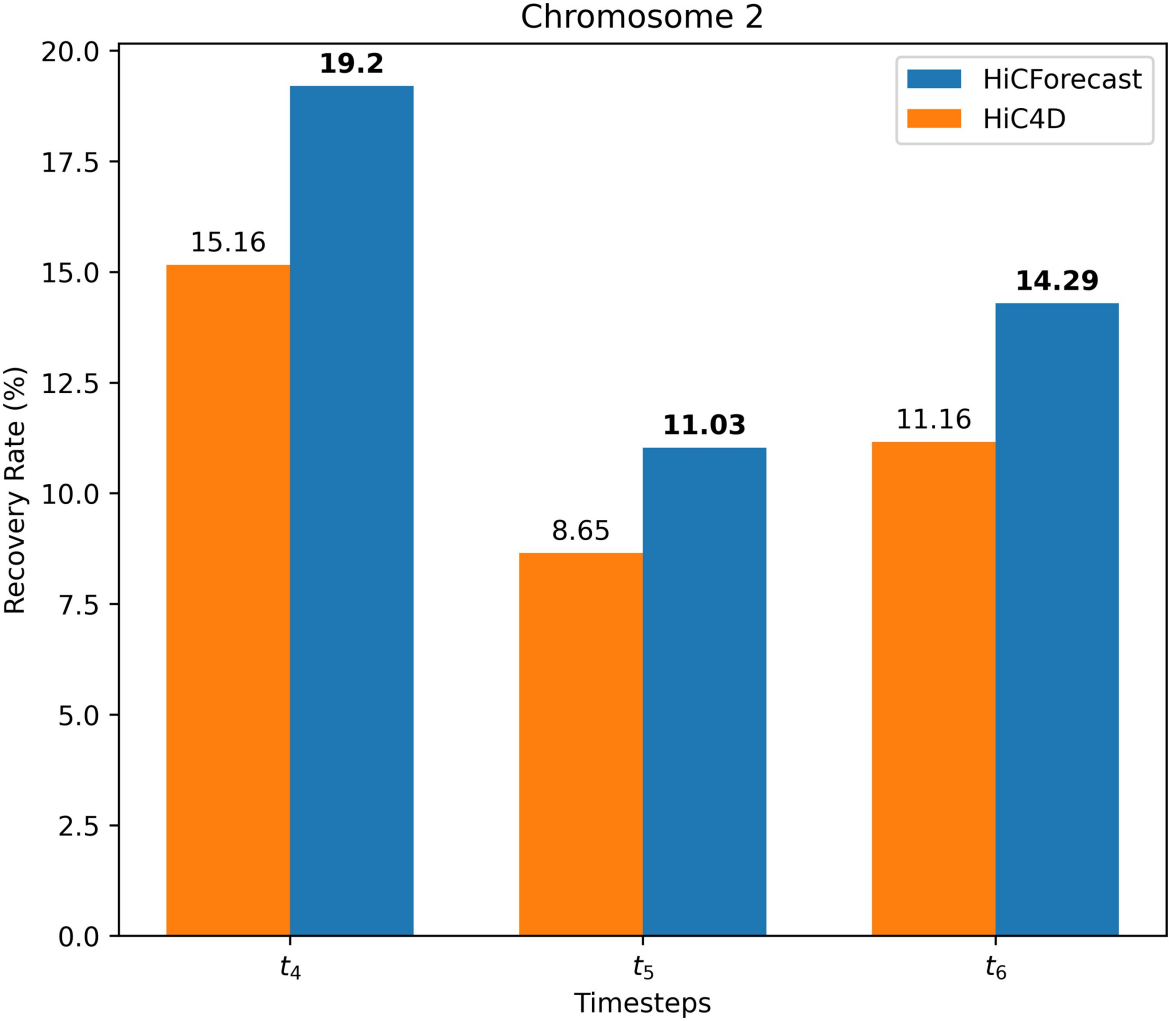
Interaction Recovery Rate using Mouse Preimplantation Embryogenesis (Dataset 1) Chromosome 2 at 40 kb resolution. Interaction recovery rate (0%–100%) indicates the recovery of interaction compared with the ground truth where HiCForecast achieved highest rate compared to HiC4D in three consecutive timesteps ($t_{4},t_{5},t_{6}$).

Both intra- and inter-chromosomal interactions are essential for important biological processes such as chromatin loop, active and inactive loci association, topological domain, etc. (Lieberman-Aiden *et al.* 2009, Dixon *et al.* 2012). We analysed the chromosomal interaction of HiCForecast and HiC4D, and calculated the interaction recovery rate. We used FitHiC (Kaul *et al.* 2020) to get the interaction in three timesteps. We fed our results into the FitHiC pipeline to produce the interactions and filtered the interaction result considering *P*-value (>.05), and produced interaction recovery rate ($=\frac{\text{target}\;\text{interaction}\;\text{count}}{\text{ground}\;\text{truth}\;\text{interaction}\;\text{count}}\times 100$) as defined and described in Chowdhury *et al.* (2024). This metric quantifies the recovery rate relative to predicted loops. The normalization is applied to prevent any method from influencing the analysis with excessive loops. We observed that HiCForecast recovers more interactions compared with HiC4D in three timesteps (Fig. 4 and Supplementary Figs S9A–S15A), which indicates that HiCForecast can preserve more chromosomal interaction information compared to HiC4D.

### 3.7 HiCForecast shows superior performance in identifying key 3D genome spatial features

**Figure 5.**
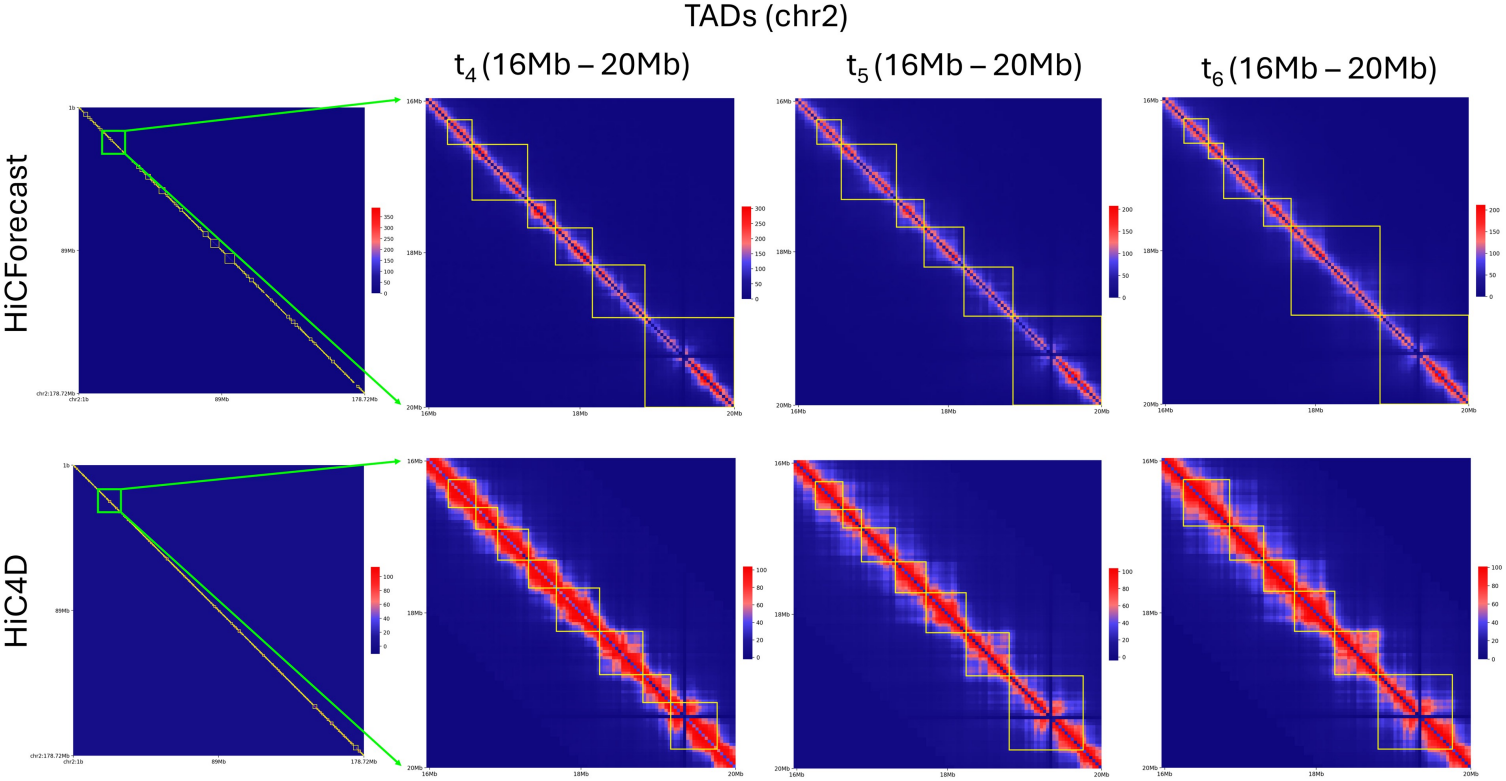
TAD region recovery using Mouse Preimplantation Embryogenesis (Dataset 1) Chromosome 2 at 40 kb resolution. TADs regions marked with squares and three consecutive timesteps are zoomed out from 16 to 20 Mb region.

Topologically associating domains (TADs) and loops are crucial spatial features in the 3D organization of the genome. TADs are regions of the genome that exhibit a higher frequency of interactions within themselves than with other regions (Dixon *et al.* 2012). Loops, on the other hand, are specific interactions between distant genomic regions that come into close spatial proximity (Rao *et al.* 2014). To account for the importance of spatial features, we used TomDom (Shin *et al.* 2016) to generate TAD regions from our result and measured measure of concordance (MoC) (Zufferey *et al.* 2018) with the ground truth to depict the percentage of TAD regions preserved by an algorithm to support the spatial feature validation. For the majority of the datasets, HiCForecast delivered the highest MoC score performance, implying a higher TAD region similarity with the ground truth (Supplementary Table S3). Figure 5 and Supplementary Figs S9B–S15B visualizes the detected TADs for a randomly selected region 16–20 Mb, TADs detected in this region are marked with squares. HiCForecast can preserve small TAD regions compared to a big region predicted by HiC4D, and this supports the capability of maintaining genome spatial features through different timesteps of HiCForecast that are present in ground truth. To further validate the biological significance, we used COVID-infected spatiotemporal Hi-C data (Zazhytska *et al.* 2022) to find differential boundaries at various timesteps (Supplementary Fig. S16 and Supplementary Section S1.2). Cresswell and Dozmorov (2020) demonstrated that different types of boundary changes reflect the underlying biology of an experimental system. In the context of the COVID-infected dataset, the observed boundary changes represent significant biological processes, providing insights into the effects of post-COVID infections. These findings further support the hypothesis that boundary changes captured in the HiCForecast data are indicative of critical biological responses to infection and can be studied to better understand the broader effects of post-COVID conditions.

### 3.8 Benchmark on 3D genome reconstruction

**Figure 6.**
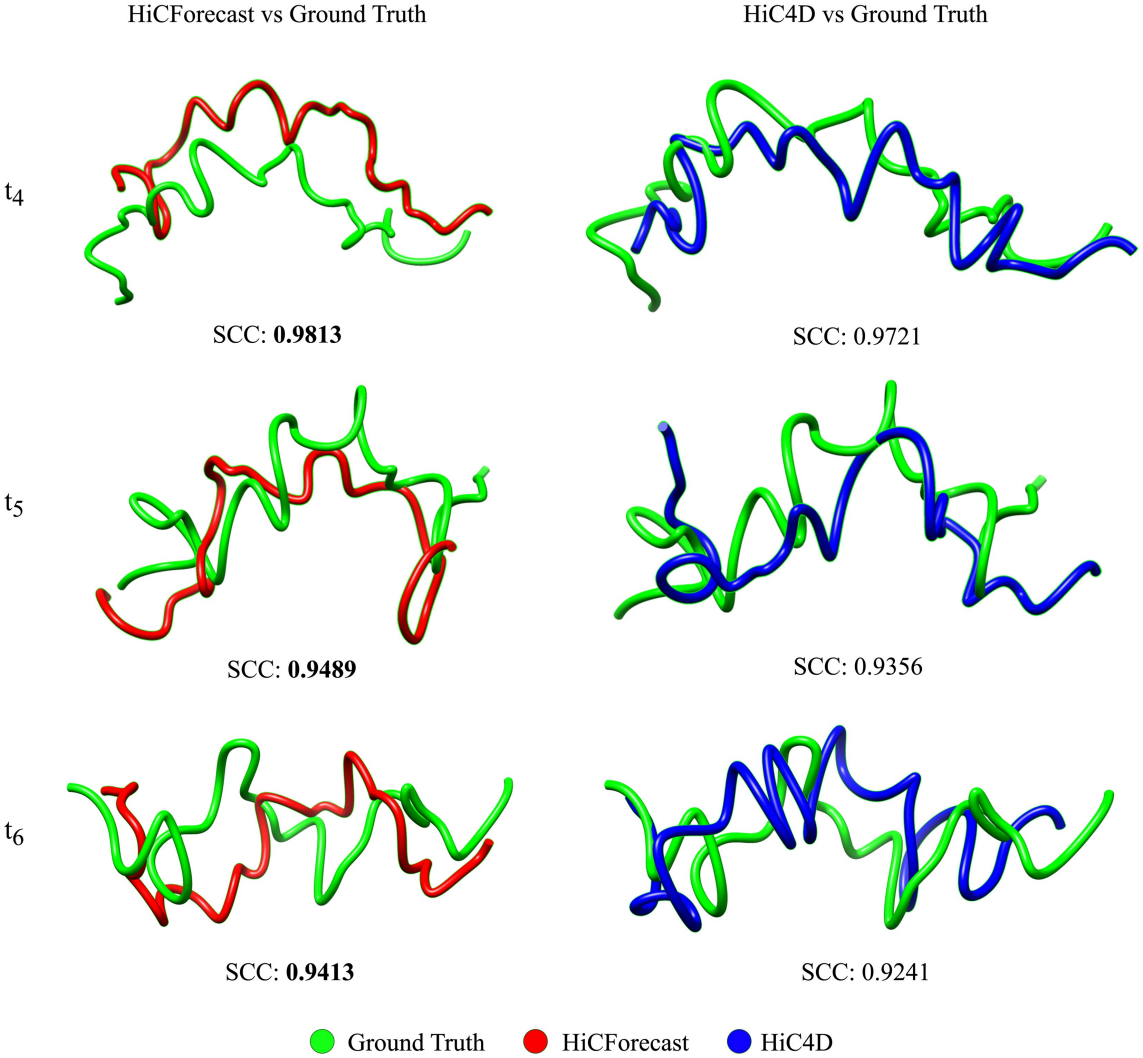
Comparison of 3D structures of chromosome 2 from Mouse Preimplantation Embryogenesis (Dataset 1) for genomic region 121 to 145 Mb. The results show that at stages 8-cell, ICM, mESC (that are $t_{4},t_{5},t_{6}$ respectively), HiCForecast demonstrates better structural similarity, as per the higher SCC scores.

3D genome structure helps us understand evolutionary constraints, cell-to-cell variability, and dynamic localization of genomic regions (Rao *et al.* 2014). We compared structures generated at different timesteps by HiCForecast and HiC4D with the ground truth. In this experiment, we selected the genomic region spanning 121–145 Mb for similarity comparison across different timesteps between the 3D structures of chromosomes from Mouse Preimplantation Embryogenesis (Dataset 1), Human Embryogenesis (Dataset 3), and Mouse Cell Reprogramming (Dataset 4)—representing each generalization category. We used 3DMax algorithm by Oluwadare *et al.* (2018) for the 3D chromosome structure reconstruction. The structural similarity across different timesteps was measured using Spearman’s correlation coefficient (SCC). We used Chimera (EF *et al.* 2004) to visualize the reconstructed 3D structures at three timesteps ($t_{4},t_{5},t_{6}$) and calculated SCC to measure structural similarity. HiCForecast predictions showed high similarity with the ground truth structures, demonstrating its effectiveness in accurately predicting timeseries chromosomal interaction data (Fig. 6 and Supplementary Figs S17–S21).

## 4 Conclusion

In this study, we introduce HiCForecast, an advanced framework for forecasting spatiotemporal Hi-C timeseries data. By employing dynamic optical flow estimation, HiCForecast effectively models complex evolutionary relationships. Utilizing the DMVFN model with a routing module for dynamic input refinement, the framework was trained on the Mouse Preimplantation Embryogenesis dataset and validated across various chromosomes, species, and biological processes. HiCForecast outperformed existing models, demonstrating superior interaction recovery, improved TAD region delineation, and closer structural similarity to ground truth. This highlights HiCForecast’s robustness in forecasting spatiotemporal Hi-C data across diverse biological contexts, demonstrating its potential for driving biological discoveries, even when only limited timeseries Hi-C data is available.

## Supplementary Material

btaf030_Supplementary_Data

## Data Availability

The datasets used in this study are GEO GSE82185 Mouse Preimplantation Embryogenesis (Du *et al.* 2017) (Dataset 1), GSA PRJCA000241 Mouse Embryogenesis (Ke *et al.* 2017) (Dataset 2), GSA CRA000852 Embryogenesis (Chen *et al.* 2019) (Dataset 3), GEO GSE96611 Mouse Cell Reprogramming (Stadhouders *et al.* 2018) (Dataset 4), NCBI BioProject PRJDB7492 Medaka Gastrulation (Nakamura *et al.* 2021) (Dataset 5), and NCBI BioProject PRJNA606649 Xenopus Tropicalis Embryogenesis (Niu *et al.* 2021) (Dataset 6). The data used in this study are publicly available for download at https://doi.org/10.5281/zenodo.14531695. HiCForecast code is publicly available at https://github.com/OluwadareLab/HiCForecast.
