# Supplementary material for: HiCForecast: dynamic network optical flow estimation algorithm for spatiotemporal Hi-C data forecasting
Source: Bioinformatics. 2025 Jan 22;41(2):btaf030. doi: 10.1093/bioinformatics/btaf030 (PMC11793695; doi:10.1093/bioinformatics/btaf030)
Supplement: btaf030_Supplementary_Data [file btaf030_supplementary_data.zip › 811a9_HiCForecast__Supplementary_Bioinformatics_Revision.pdf]

SUPPLEMENTARY MATERIAL

HiCForecast: Dynamic Network Optical Flow Estimation Algorithm for Spatiotemporal Hi-C Data Forecasting

Dmitry Pinchuk,[1] H. M. A. Mohit Chowdhury,[2] Abhishek Pandeya[2] and Oluwatosin Oluwadare[2,3,*]

[1]Department of Computer Science, University of Wisconsin-Madison, 500 Lincoln Dr, 53706, WI, USA, [2]Department of Computer Science, University of Colorado at Colorado Springs, 1420 Austin Bluffs Pkwy, Colorado Springs, 80918, Colorado, USA and [3]Department of Biomedical Informatics, University of Colorado Anschutz Medical Campus, 13001 East 17th Place, Aurora, 80045, Colorado, USA
*Corresponding Author: ooluwada@uccs.edu
FOR PUBLISHER ONLY Received on Date Month Year; revised on Date Month Year; accepted on Date Month Year

1. Results

1.1 Generalization to Different Species

1.1.1 Medaka Gastrulation (Dataset 5)

HiCForecast showed a consistently stronger performance than HiC4D on the Medaka gastrulation dataset across all evaluation metrics for all timesteps on both tested chromosomes (Supplementary Table S2). This provides further evidence of the ability of HiCForecast to generalize to embryogensis datasets of other species. Additionally, the comparison between different timesteps within the predicted timeseries of this dataset is available in Supplementary Figure S7.

1.1.2 Xenopus Tropicalis Embryogenesis (Dataset 6)

This dataset of Xenopus tropicalis embryogenesis has the same system but different species from the training Mouse Preimplantation Embryogenesis dataset. HiCForecast delivers a robust result consistently outperforming HiC4D across all timesteps in all evaluation metrics (Supplementary Table S2). These results further indicate the ability of HiCForecast to generalize to embryogenesis datasets of other species. Additionally, the comparison between different timesteps within the predicted timeseries of this dataset is available in Supplementary Figure S8.

1.2 HiCForecast detects differential boundaries at various timesteps using COVID-infected spatiotemporal Hi-C data.

Observing interacting boundary change is crucial for understanding biological functionality such as neural development, cellular development process, etc. These boundary changes are measured for a particular timestep compared with the earlier timestep to observe how they change their structure over time. These types of interacting boundary changes over time establish the demand for future timestep data to further investigate biological functionality. Cresswell et al. [1] categorized domain changes into five categories: Merge, Split, Complex, Shifted, and Strength Change.

To analyze the interacting boundary change over time, we utilized Hamster COVID-infected dataset [2]. The experiment conducted by Zazhytska et al. [2] utilized Hamster infected by SARS-CoV-2, which yields a Hi-C timeseries of cells 12.5 hours post infection, and 1, 3 and 10 days post infection (dpi) as well as a non-infected control cell. First, we used control and 1dpi data as input in HiCForecast to predict a future timestep (Figure S16A). Second, we used 1dpi and 3dpi as input in HiCForecast and predicted a future timestep (Figure S16B). Next, to demonstrate the biological significance of Hi-C data forecasts made by HiCForecast, we then analyzed the interacting boundary changes that occur in future predictions compared to previous timestep.

Figure S16C and S16D show the proportion of boundary changes observed in the entire matrix for Figure S16A and Figure S16B respectively. For a specific region 16 MB to 20 MB, in Figure S16A, the upper heatmap represents 1dpi and the lower heatmap region represents the HiCForecast (future timestep) data of chromosome 6. In Figure S16B, the upper heatmap represents 3dpi and the lower heatmap region represents the HiCForecast (future timestep) data of chromosome 6. We observed three types of boundary change (Figure S16A) and four types of boundary change (Figure S16B) between these two timesteps. This change is calculated with the differential boundary score shown in the bottom-line plot. This score is centered on 0 and boundary changes are detected using the absolute differential boundary score > 2 [1].

Cresswell et al. [1] demonstrated that different types of boundary changes reflect the underlying biology of an experimental system. In the context of the COVID-infected dataset, the observed boundary changes represent significant biological processes, providing insights into the effects of post-COVID infections. For instance, Zazhytska et al. [2] highlighted notable compartmental changes in olfactory receptors (ORs) following COVID infection, emphasizing the importance of these structural alterations. These findings further support the hypothesis that boundary changes captured in the HiCForecast data are indicative of critical biological responses to infection and can be studied to better understand the broader effects of post-COVID conditions.

Table 1. Various hyperparameter combinations considered in the hyperparameter search. The best model, which is selected to be HiCForecast, is highlighted in green.

Loss Function	window size	batch size	learning rate	normalization	id
L1 Pyramidal + VGG	64	8	default (cos annealing)	250	
MSE + VGG	64	8	default (cos annealing)	250	
MSE	64	8	default (cos annealing)	250	
L1	64	8	default (cos annealing)	250	model 1
L1 Pyramidal	64	8	default (cos annealing)	250	model 2
L1 + VGG	64	8	default (cos annealing)	250	
L1 Pyramidal + VGG	96	8	default(cos annealing)	250	
MSE	96	8	default(cos annealing)	250	
L1	96	8	default(cos annealing)	250	model 3
L1 Pyramidal	96	8	default(cos annealing)	250	
MSE + VGG	96	8	default(cos annealing)	250	
L1	48	8	default(cos annealing)	250	
MSE + VGG	48	8	default(cos annealing)	250	
MSE	48	8	default(cos annealing)	250	
L1 + VGG	48	8	default(cos annealing)	250	
L1	128	8	default(cos annealing)	250	model 4
L1	48	8	default (cos annealing)	100	
L1	48	8	default (cos annealing)	150	
L1	48	8	default (cos annealing)	200	
L1	64	8	default (cos annealing)	100	
L1	64	8	default (cos annealing)	150	
L1	48	8	default (cos annealing)	300	
L1	48	8	default (cos annealing)	max	
L1	64	8	default (cos annealing)	max	
L1	64	8	default (cos annealing)	300	HiCForecast
L1	48	32	default (cos annealing)	250	
L1	48	64	default (cos annealing)	250	
L1	48	128	default (cos annealing)	250	
L1	64	32	default (cos annealing)	250	
L1	64	64	default (cos annealing)	250	
L1	48	8	default (cos annealing)	400	
L1	48	8	default (cos annealing)	500	model 5
L1	48	8	const. 1e-3	250	
L1	48	8	const. 1e-5	250	
L1	48	8	const. 1e-4	250	
L1	48	32	4*default (cos annealing)	250	
L1	48	64	8*default (cos annealing)	250	
L1	48	8	1e-3 to 1e-5 cos annealing	250	
L1	48	128	16*default (cos annealing)	250	
L1	48	128	4*default (cos annealing)	250	
L1	64	8	1.00E-04	300	
L1	64	8	1.50E-03	300	
L1	64	8	1.50E-04	300	
L1	64	32	4*default (cos annealing)	300	
L1	64	8	default(cos annealing)	350	
L1	64	8	default(cos annealing)	400	model 6

Table 2. Test results of HiCForecast and HiC4D for predicting timesteps 4, 5 and 6 on chromosomes 2 and 6 of datasets 5 and 6 evaluated with GenomeDISCO, PCC and PSNR metrics. Chromosomes 2 and 6 are the blind test chromosomes of Mouse Preimplantation Embryogenesis (Dataset 1), which was used for training. The results on datasets 2 to 6 are blind generalizations test on different species and systems. The bold highlight indicates a higher score compared to the other method for the given future time points.

			t_4		t_5		t_6	
			HiCForecast	HiC4D	HiCForecast	HiC4D	HiCForecast	HiC4D
Medaka Gastrulation - Dataset 5 [3]	chr 2	GenomeDISCO	**0.814**	0.718	**0.739**	0.657	**0.746**	0.688
		PCC	**0.937**	0.792	**0.923**	0.824	**0.892**	0.801
		PSNR	**34.372**	28.588	**32.919**	28.383	**34.375**	30.528
	chr 6	GenomeDISCO	**0.748**	0.632	**0.687**	0.597	**0.710**	0.661
		PCC	**0.919**	0.776	**0.905**	0.810	**0.879**	0.787
		PSNR	**27.758**	22.904	**27.173**	22.788	**29.236**	24.961
Xenopus Tropicalis Embryogenesis - Dataset 6 [4]	chr 2	GenomeDISCO	**0.839**	0.743	**0.803**	0.678	**0.728**	0.696
		PCC	**0.972**	0.883	**0.956**	0.917	**0.931**	0.893
		PSNR	**36.257**	28.116	**33.898**	29.286	**33.795**	31.633
	chr 6	GenomeDISCO	**0.849**	0.743	**0.809**	0.693	**0.727**	0.705
		PCC	**0.972**	0.885	**0.956**	0.920	**0.932**	0.894
		PSNR	**36.583**	28.705	**34.371**	29.683	**32.562**	30.591

Table 3. Measure of Concordance (MoC) using dataset 1, 2, 3, 4 of chromosome 2 and 6 at 40kb resolution. This score (0 = totally different, 1 = totally similar) shows how much TAD regions structural similarity preserved in a particular algorithm's prediction compared with the ground truth across three consecutive timesteps (t_4, t_5, t_6).

Dataset	Chromosome	Timestep	HiCForecast	HiC4D
1	2	8-cell (t_4)	**0.69**	0.58
		ICM (t_5)	**0.7**	0.6
		mESC (t_6)	**0.69**	0.58
	6	8-cell (t_4)	**0.72**	0.59
		ICM (t_5)	**0.66**	0.63
		mESC (t_6)	**0.68**	0.6
2	2	8-cell (t_4)	0.7	**0.71**
		E3.5(t_5)	0.66	**0.7**
		E7.5(t_6)	0.66	**0.67**
	6	8-cell(t_4)	0.72	**0.74**
		E3.5(t_5)	0.66	**0.71**
		E7.5(t_6)	0.65	**0.68**
3	2	blastocyst (t_4)	**0.67**	0.59
		6-week (t_5)	**0.63**	0.61
		hESC (t_6)	**0.64**	0.6
	6	blastocyst(t_4)	**0.68**	0.63
		6-week (t_5)	**0.65**	0.63
		hESC (t_6)	**0.64**	0.63
4	2	D4 (t_4)	**0.88**	0.75
		D6 (t_5)	**0.83**	0.74
		D8 (t_6)	**0.79**	0.71
	6	D4 (t_4)	**0.86**	0.76
		D6 (t_5)	**0.8**	0.76
		D8 (t_6)	**0.76**	0.73

Table 4. SSIM index scores for Dataset 2, 3 and 4 at 40kb resolution. SSIM index in the range $0-1$, where high is better, compares the structural similarity between predicted and ground truth maps by measuring structural preservation. HiCForecast achieved the highest score in most cases across the timesteps.

Dataset	Chromosome	Timestep	HiCForecast	HiC4D
2	2	8-cell (t_4)	**0.9799**	0.9266
		E3.5(t_5)	**0.9799**	0.9088
		E7.5(t_6)	**0.9712**	0.9160
	6	8-cell(t_4)	**0.9723**	0.9065
		E3.5(t_5)	**0.9727**	0.8908
		E7.5(t_6)	**0.9553**	0.8975
3	2	blastocyst (t_4)	0.9629	**0.9722**
		6-week (t_5)	**0.9469**	0.9464
		hESC (t_6)	**0.9365**	0.9025
	6	blastocyst(t_4)	**0.9602**	0.9579
		6-week (t_5)	0.9448	**0.9457**
		hESC (t_6)	0.9255	**0.9342**
4	2	D4 (t_4)	**0.9307**	0.8060
		D6 (t_5)	**0.9339**	0.8319
		D8 (t_6)	**0.9128**	0.8073
	6	D4 (t_4)	**0.9072**	0.7637
		D6 (t_5)	**0.9139**	0.8098
		D8 (t_6)	**0.8894**	0.7908

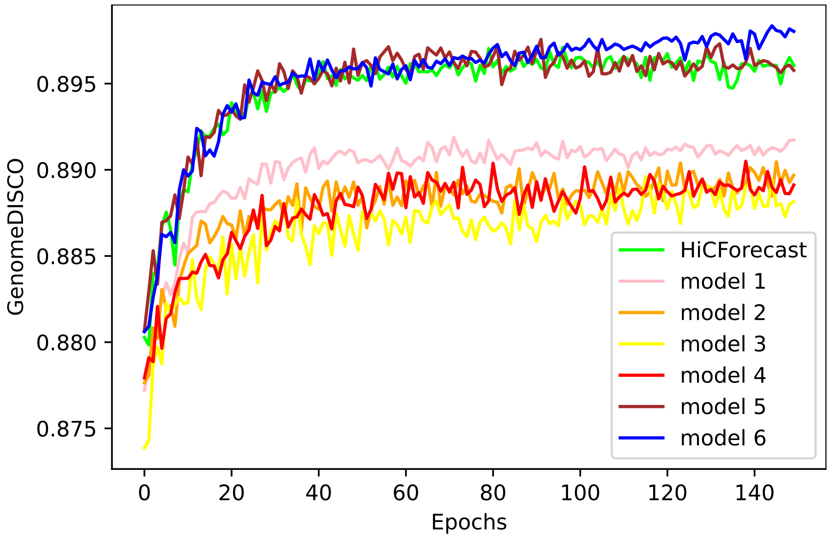

Fig. 1. Validation comparison of the highest performing models for predicting timestep 4 on the validation chromosome dataset 19 of dataset 1. The model configurations are denoted in Table 1.

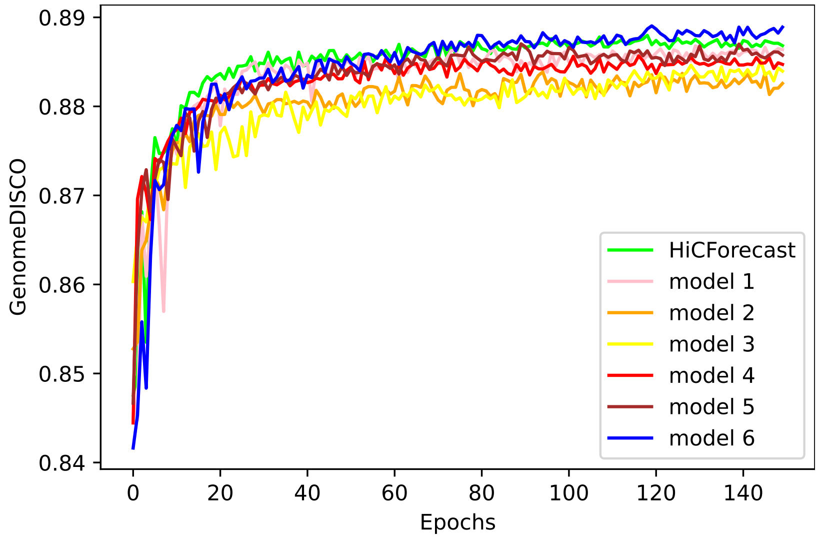

Fig. 2. Validation comparison of the highest performing models for predicting timestep 5 on the validation chromosome dataset 19 of dataset 1. The model configurations are denoted in Table 1.

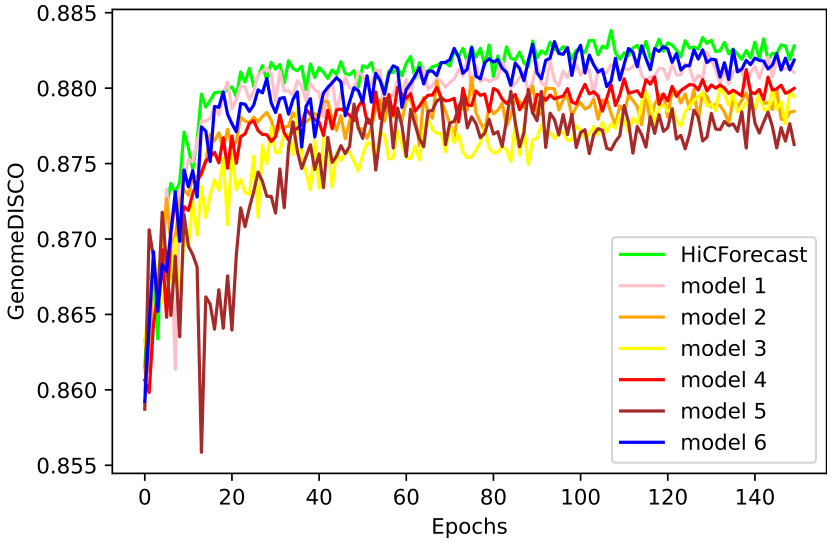

Fig. 3. Validation comparison of the highest performing models for predicting timestep 6 on the validation chromosome dataset 19 of dataset 1. The model configurations are denoted in Table 1.

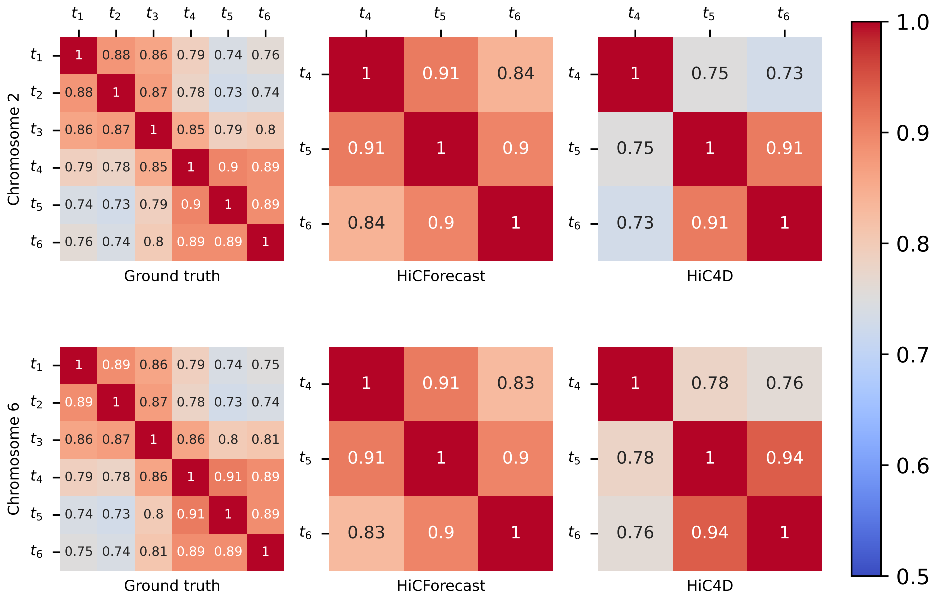

Fig. 4. GenomeDISCO scores between ground truth timesteps 1-6 and the predictions of HiCForecast and HiC4D on dataset 2 chromosomes 2 and 6 demonstrating the similarity between different timesteps for both ground truth and predicted Hi-C timeseries.

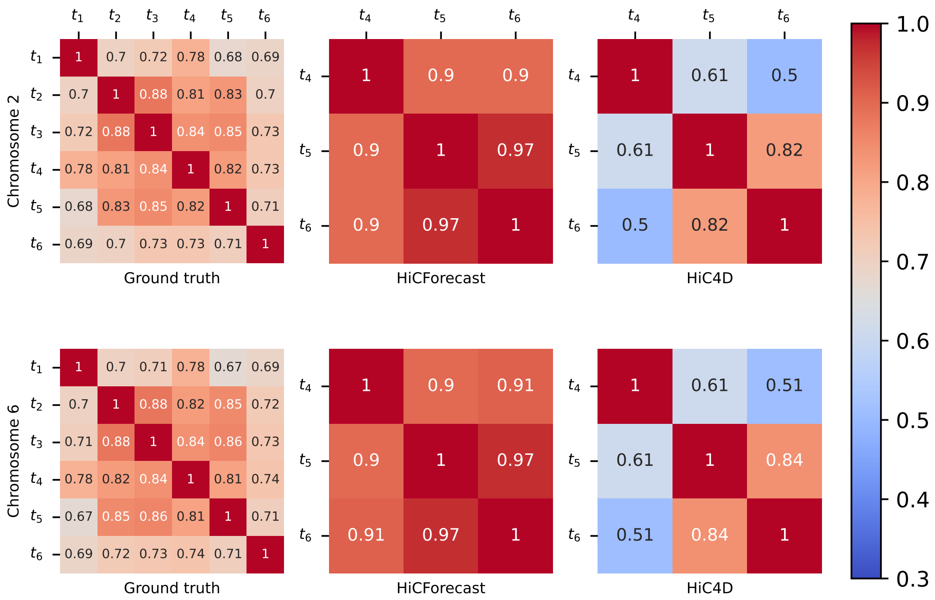

Fig. 5. GenomeDISCO scores between ground truth timesteps 1-6 and the predictions of HiCForecast and HiC4D on dataset 3 chromosomes 2 and 6 demonstrating the similarity between different timesteps for both ground truth and predicted Hi-C timeseries. Note that the HiC4D predictions for this dataset have been adjusted by setting any negative values to 0, as the original predictions included some negative values.

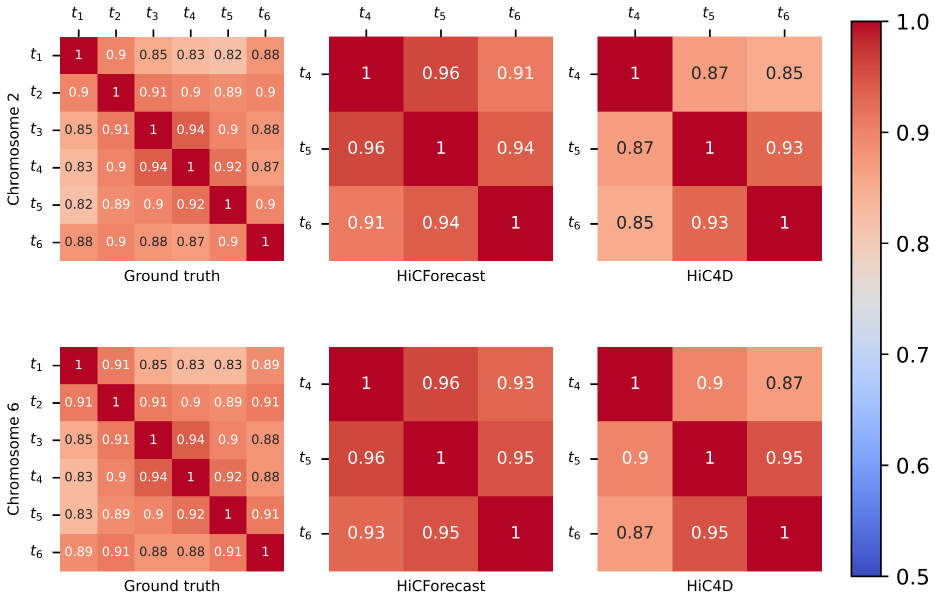

Fig. 6. GenomeDISCO scores between ground truth timesteps 1-6 and the predictions of HiCForecast and HiC4D on dataset 4 chromosomes 2 and 6 demonstrating the similarity between different timesteps for both ground truth and predicted Hi-C timeseries.

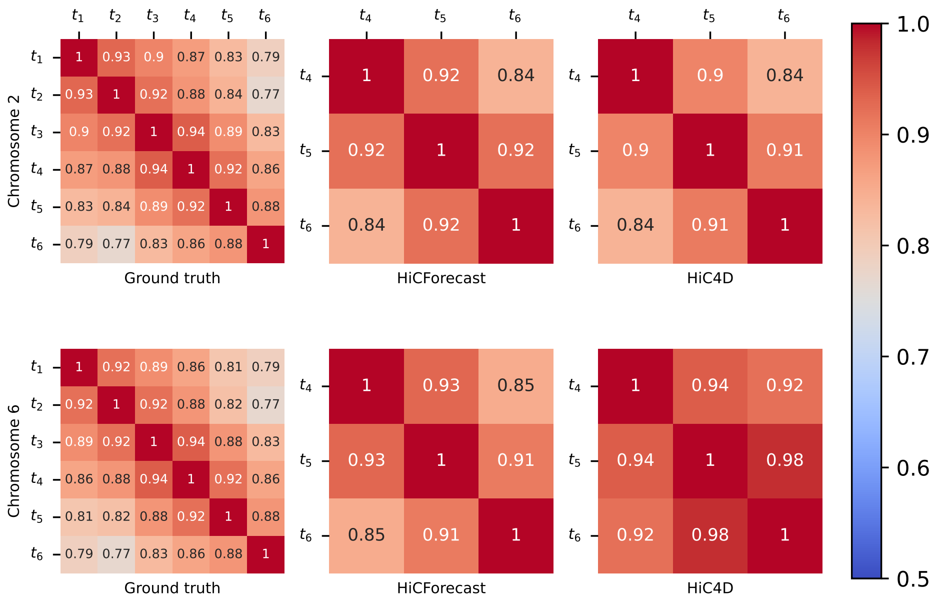

Fig. 7. GenomeDISCO scores between ground truth timesteps 1-6 and the predictions of HiCForecast and HiC4D on dataset 5 chromosomes 2 and 6 demonstrating the similarity between different timesteps for both ground truth and predicted Hi-C timeseries.

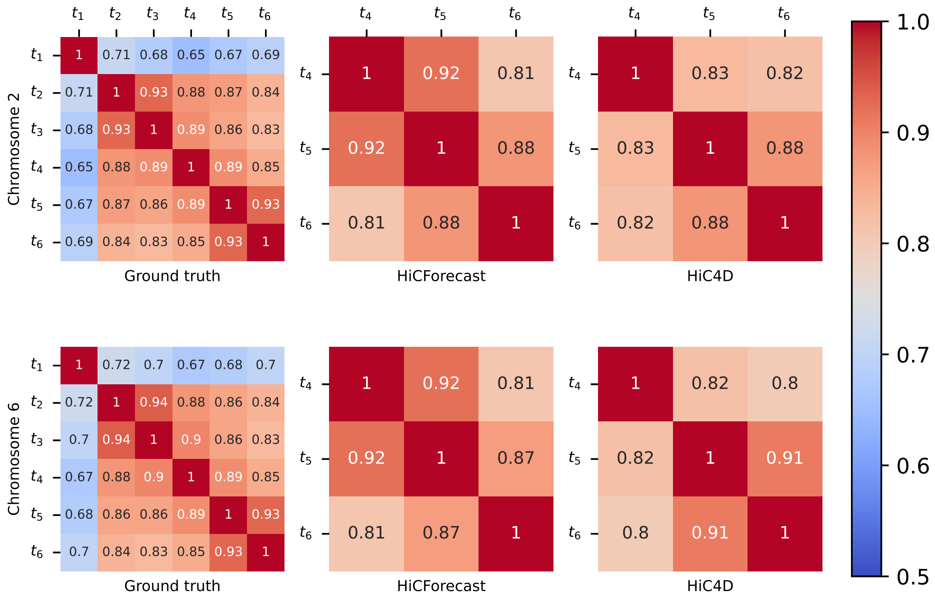

Fig. 8. GenomeDISCO scores between ground truth timesteps 1-6 and the predictions of HiCForecast and HiC4D on dataset 6 chromosomes 2 and 6 demonstrating the similarity between different timesteps for both ground truth and predicted Hi-C timeseries.

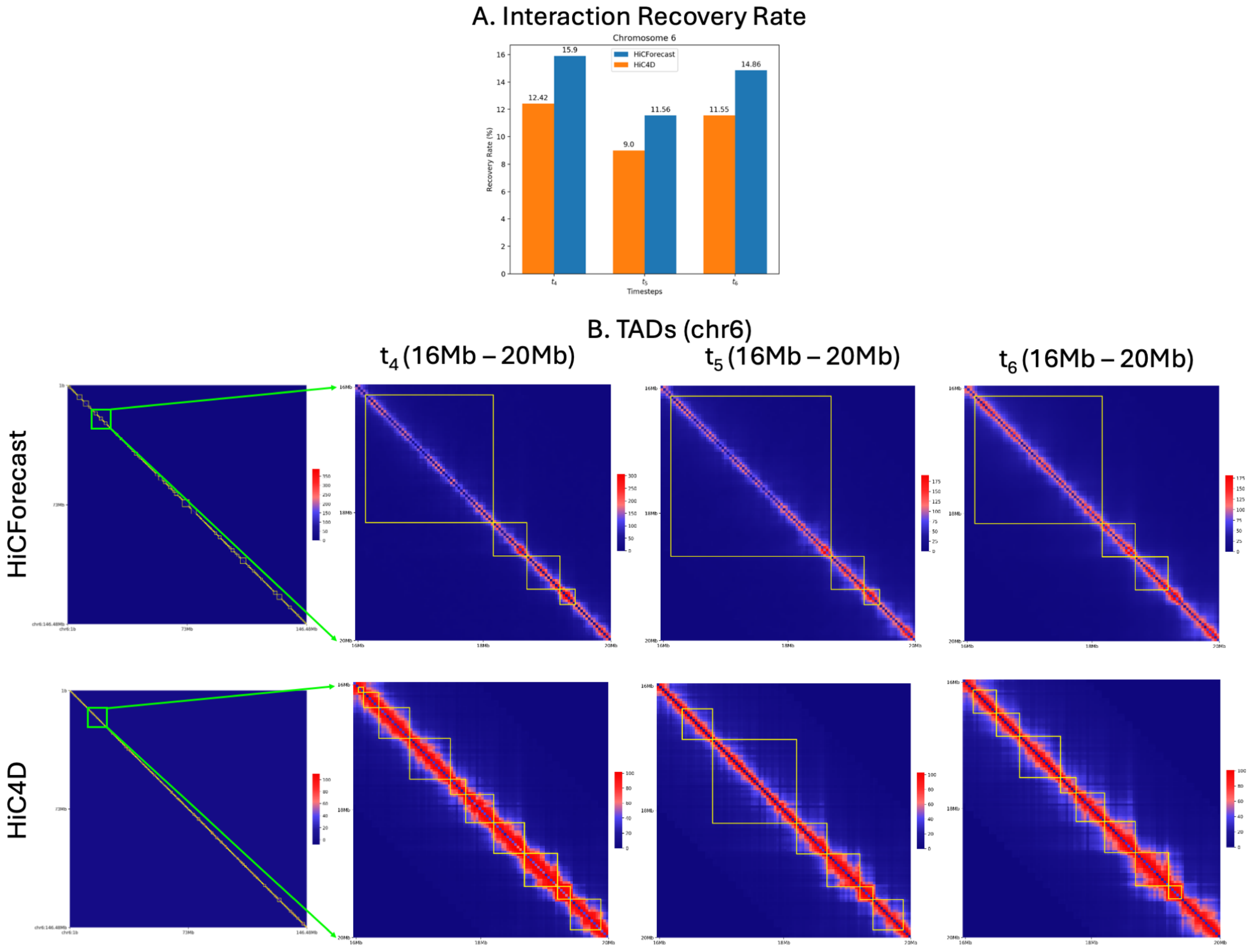

Fig. 9. Biological feature validation using Dataset 1 (Mouse Preimplantation Embryogenesis) Chromosome 6 at 40kb resolution. A. Interaction Recovery Rate ($0 - 100\%$) indicates the recovery of interaction compared with the ground truth where HiCForecast achieved highest rate compared to HiC4D in three consecutive timesteps (t_4, t_5, t_6), B. visualization of TADs (yellow rectangle) region in between 16Mb to 20Mb across three consecutive timesteps.

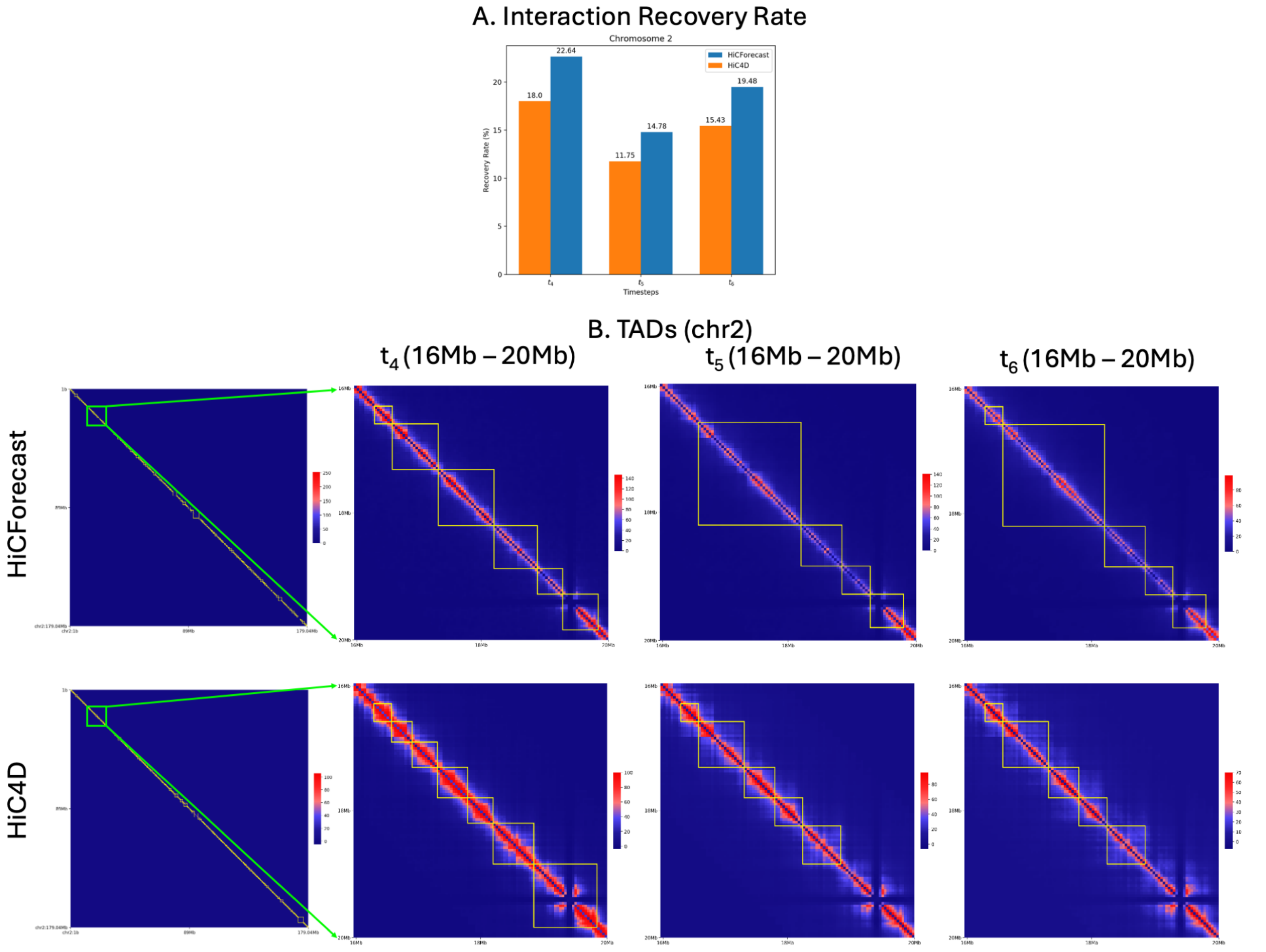

Fig. 10. Biological feature validation using Mouse Embryogenesis (Dataset 2) Chromosome 2 at 40kb resolution. A. Interaction Recovery Rate ($0-100\%$) indicates the recovery of interaction compared with the ground truth where HiCForecast achieved highest rate compared to HiC4D in three consecutive timesteps (t_4, t_5, t_6), B. visualization of TADs (yellow rectangle) region in between 16Mb to 20Mb across three consecutive timesteps.

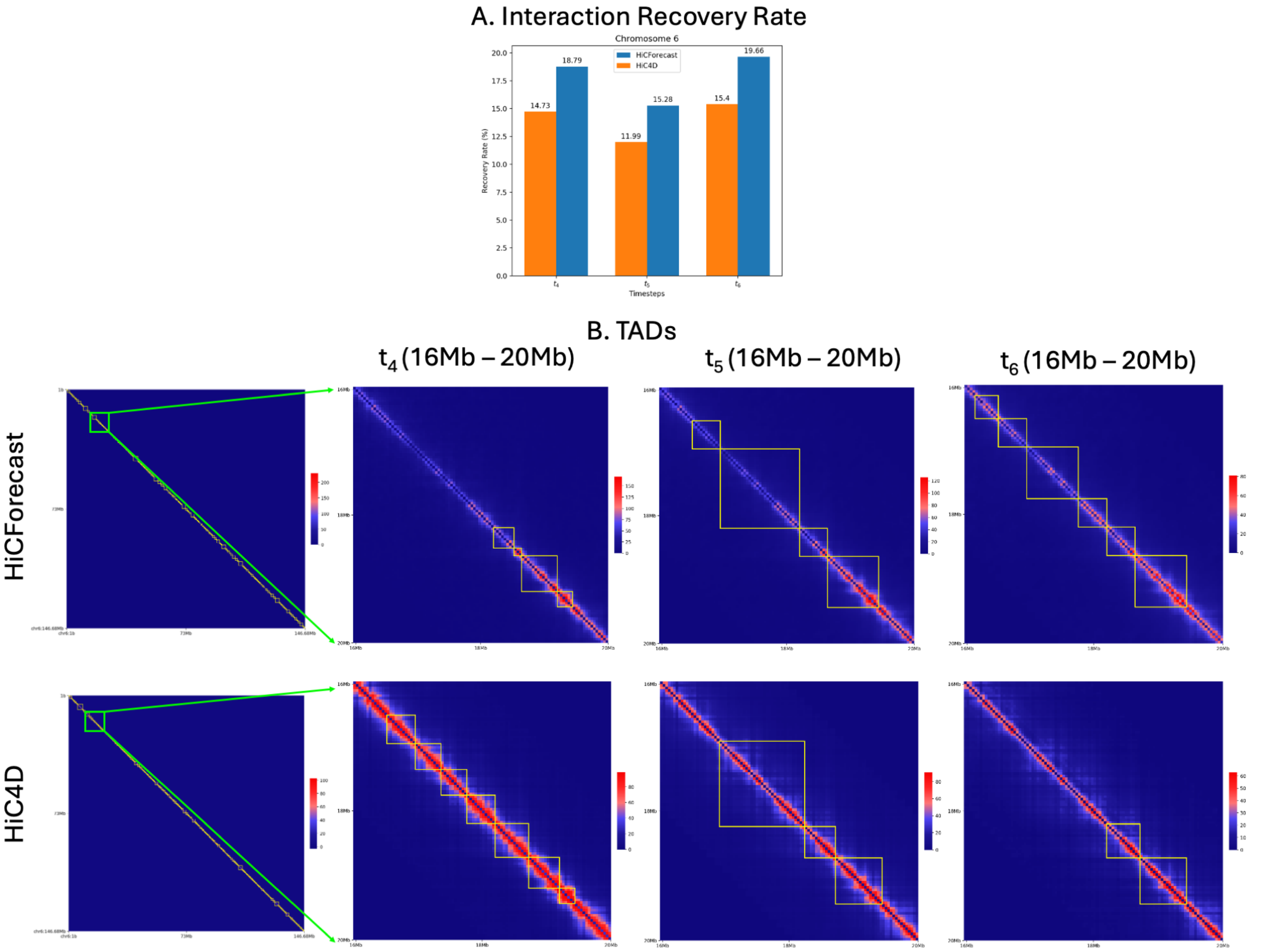

Fig. 11. Biological feature validation using Mouse Embryogenesis (Dataset 2) Chromosome 6 at 40kb resolution. A. Interaction Recovery Rate $(0-100\%)$ indicates the recovery of interaction compared with the ground truth where HiCForecast achieved highest rate compared to HiC4D in three consecutive timesteps (t_4, t_5, t_6), B. visualization of TADs (yellow rectangle) region in between 16Mb to 20Mb across three consecutive timesteps.

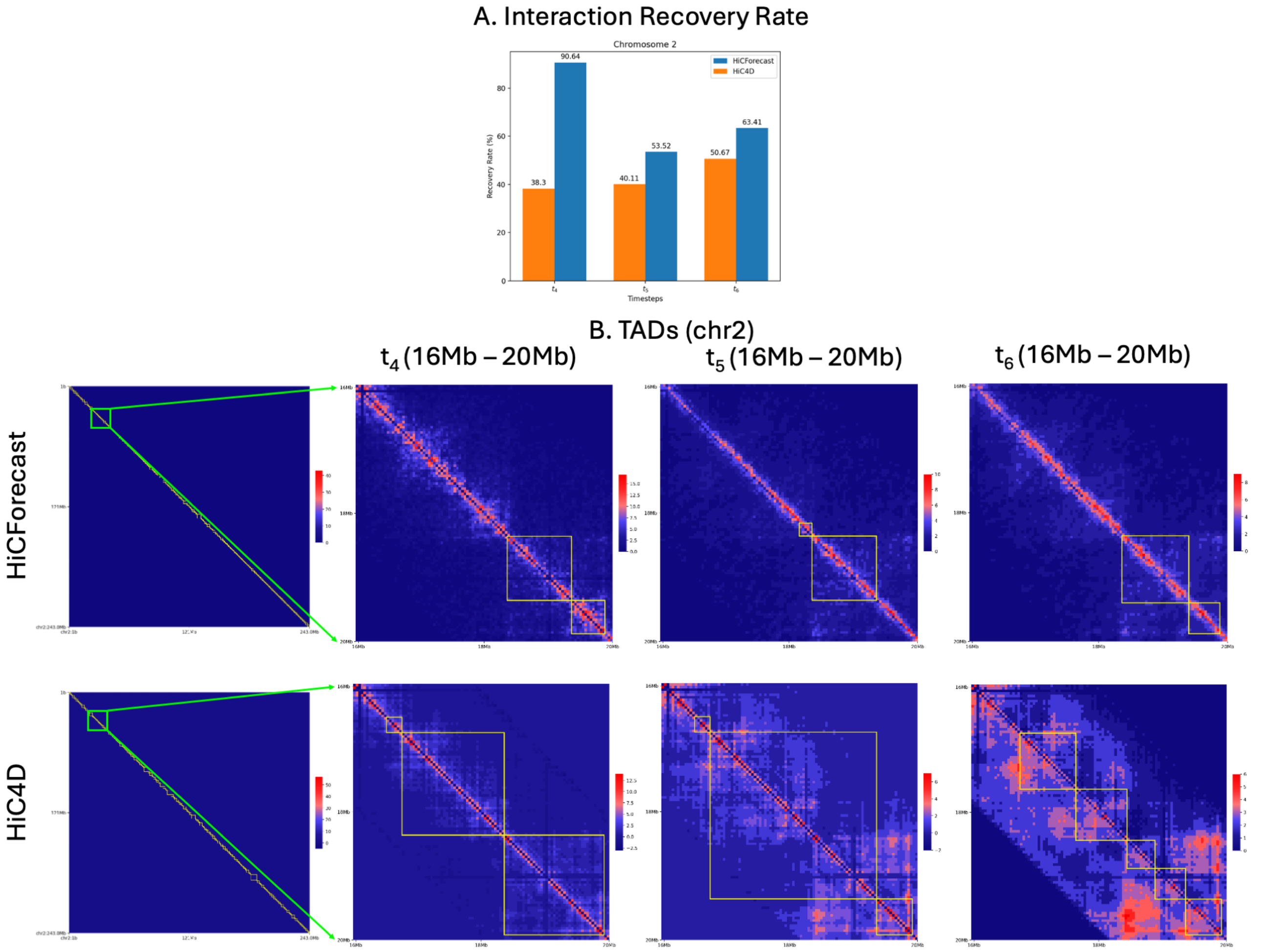

Fig. 12. Biological feature validation using Human Embryogenesis (Dataset 3) Chromosome 2 at 40kb resolution. A. Interaction Recovery Rate ($0 - 100\%$) indicates the recovery of interaction compared with the ground truth where HiCForecast achieved highest rate compared to HiC4D in three consecutive timesteps (t_4, t_5, t_6), B. visualization of TADs (yellow rectangle) region in between 16Mb to 20Mb across three consecutive timesteps.

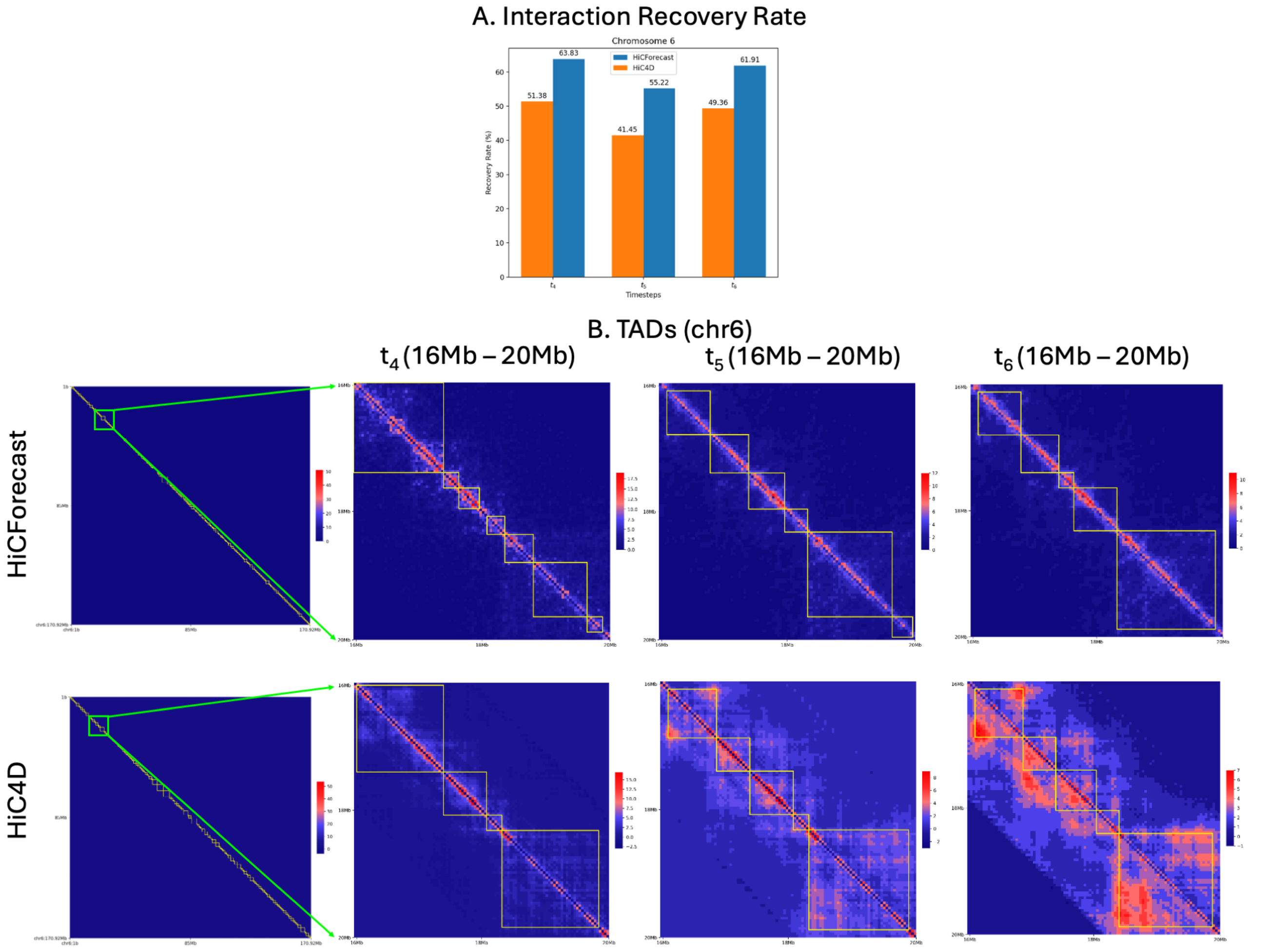

Fig. 13. Biological feature validation using Human Embryogenesis (Dataset 3) Chromosome 6 at 40kb resolution. A. Interaction Recovery Rate $(0 - 100\%)$ indicates the recovery of interaction compared with the ground truth where HiCForecast achieved highest rate compared to HiC4D in three consecutive timesteps (t_4, t_5, t_6), B. visualization of TADs (yellow rectangle) region in between 16Mb to 20Mb across three consecutive timesteps.

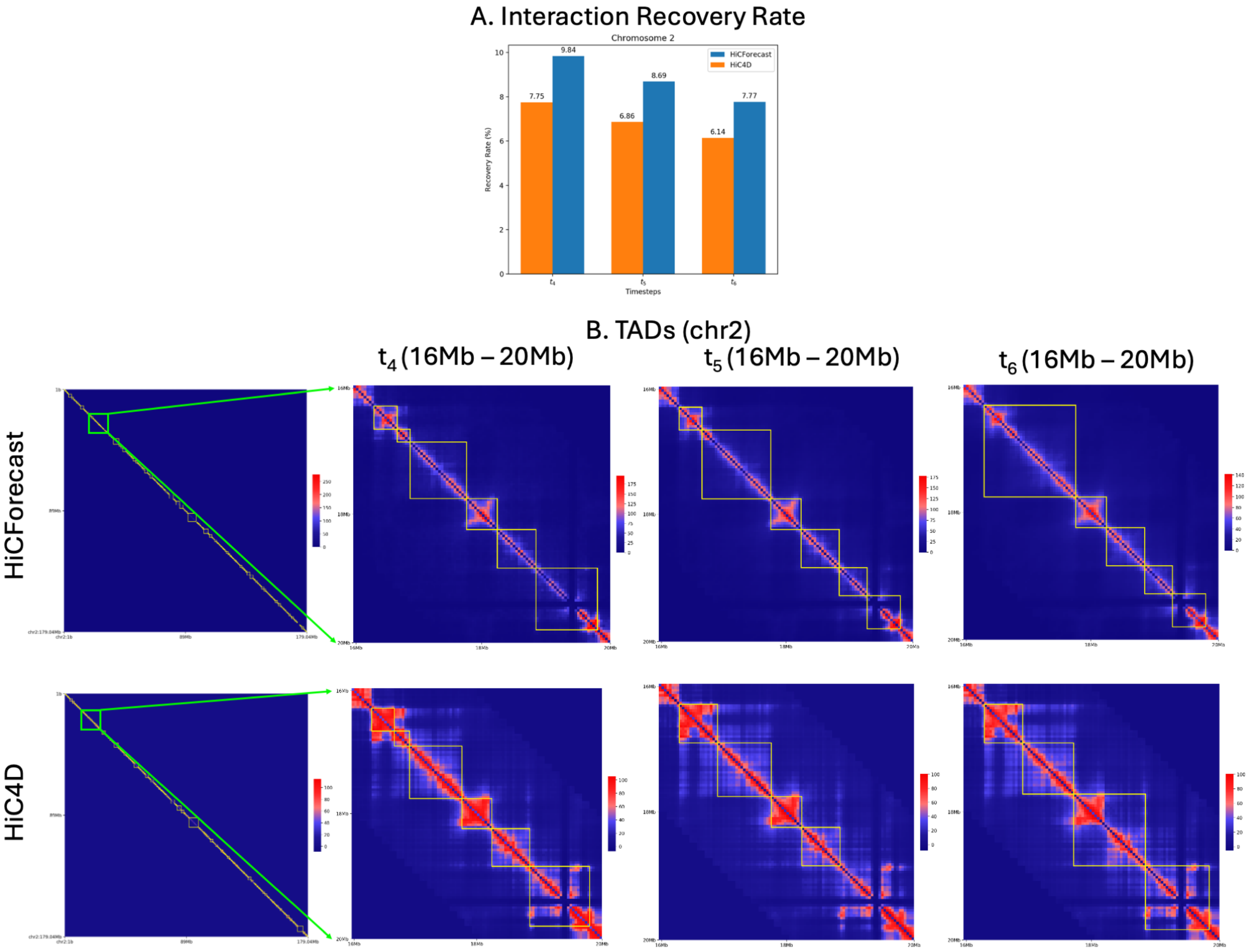

Fig. 14. Biological feature validation using Mouse Cell Reprogramming (Dataset 4) Chromosome 2 at 40kb resolution. A. Interaction Recovery Rate ($0-100\%$) indicates the recovery of interaction compared with the ground truth where HiCForecast achieved highest rate compared to HiC4D in three consecutive timesteps (t_4, t_5, t_6), B. visualization of TADs (yellow rectangle) region in between 16Mb to 20Mb across three consecutive timesteps.

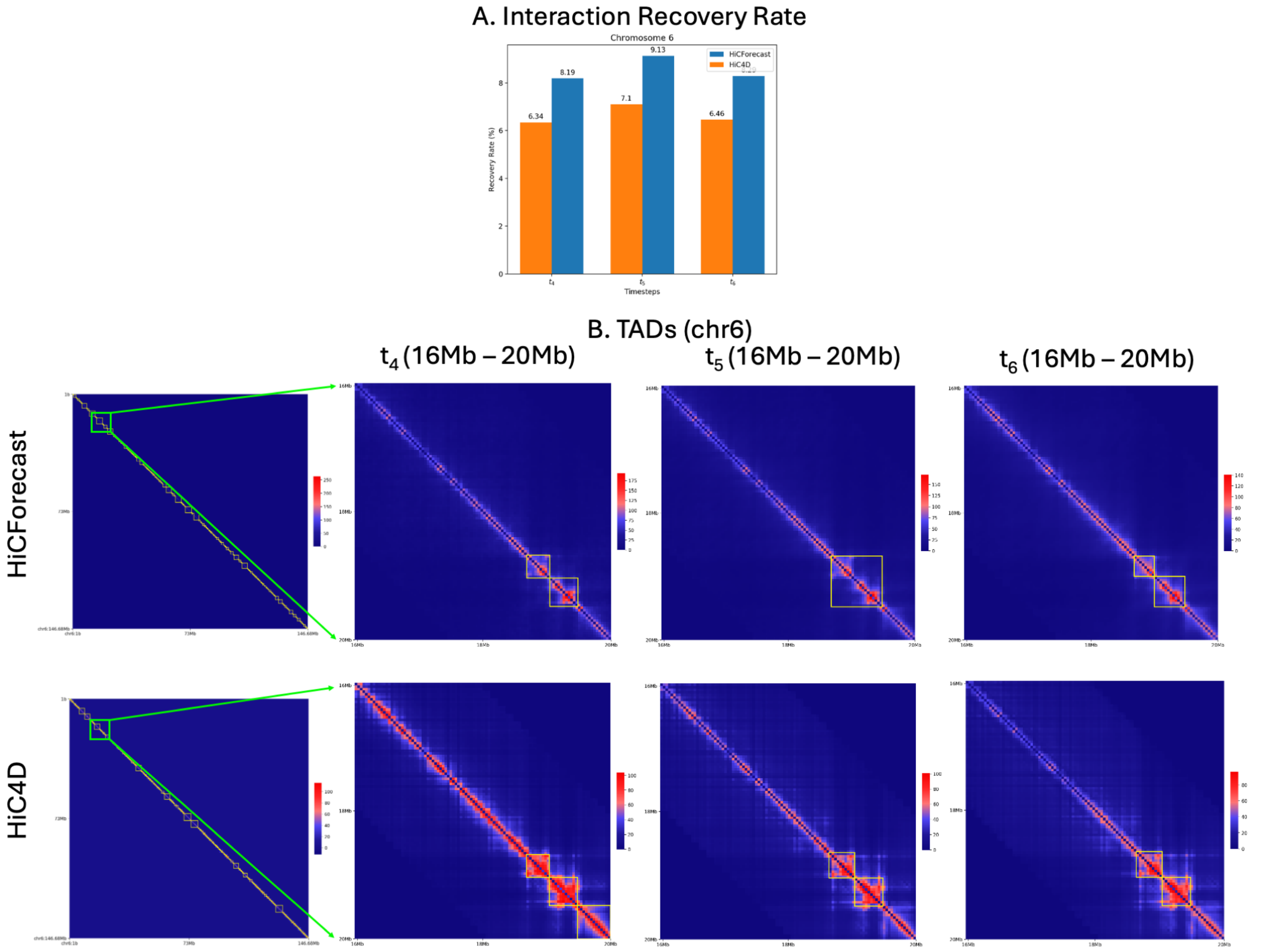

Fig. 15. Biological feature validation using Mouse Cell Reprogramming (Dataset 4) Chromosome 6 at 40kb resolution. A. Interaction Recovery Rate ($0 - 100\%$) indicates the recovery of interaction compared with the ground truth where HiCForecast achieved highest rate compared to HiC4D in three consecutive timesteps (t_4, t_5, t_6), B. visualization of TADs (yellow rectangle) region in between 16Mb to 20Mb across three consecutive timesteps.

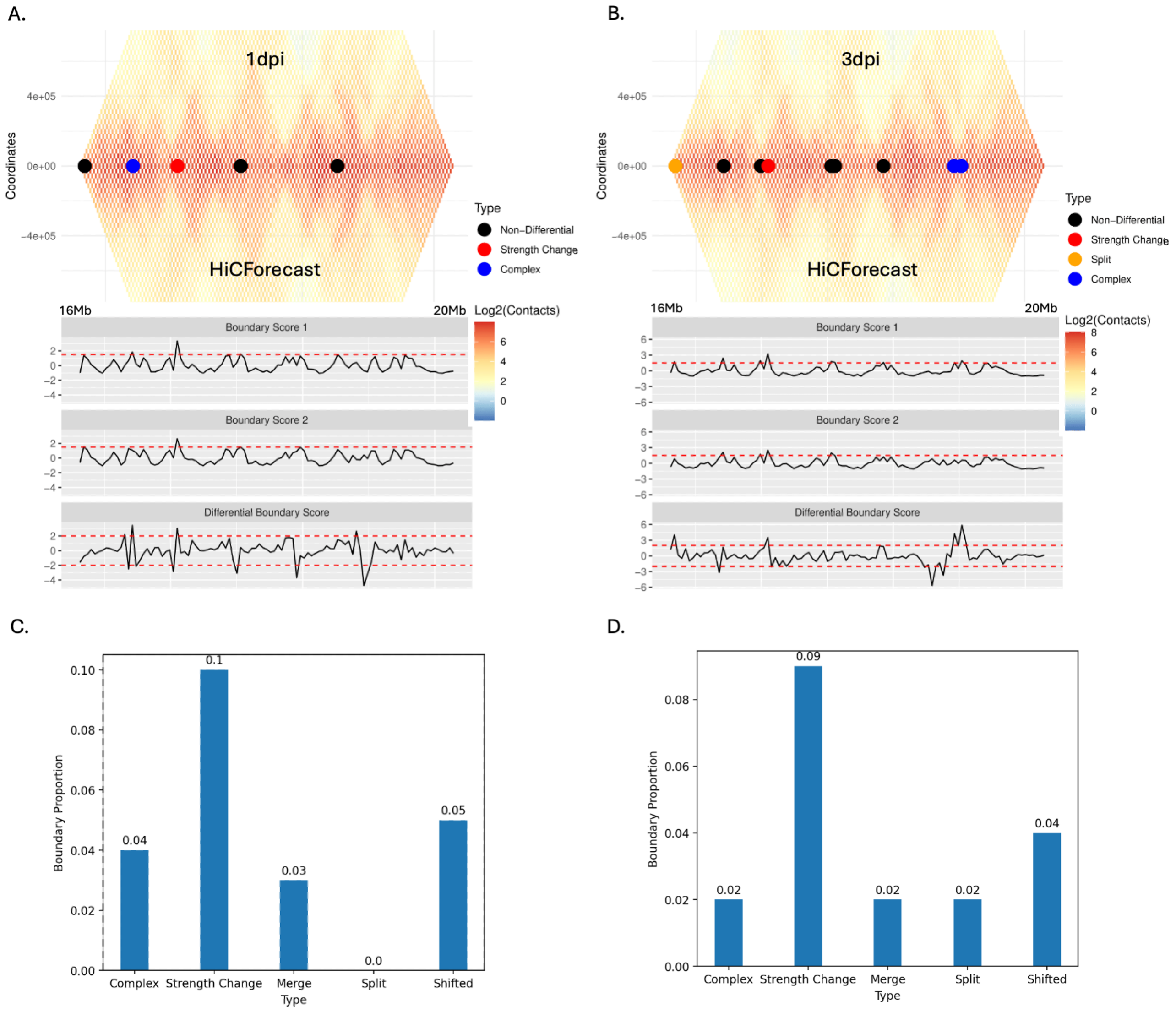

Fig. 16. Differential boundaries of chromosome 6 using Hamster COVID infected dataset. (A) 1dpi vs HiCForecast and (B) 3dpi vs HiCForecast data for 16Mb to 20Mb region. For A and B, the upper heatmap represents the previous timestep data, the lower heatmap represents HiCForecast (Future) data, and the circular marked position represents different types of boundary changes at that specific position. Boundary score 1 represents previous timestep data, Boundary Score 2 represents HiCForecast (Future), and Differential boundary score indicates the position where boundaries are changed. Boundary proportion of different types of boundary changes of chromosome 6. (C) The proportion of boundary change for 1dpi vs HiCForecast (D) The proportion of boundary change for 3dpi vs HiCForecast.

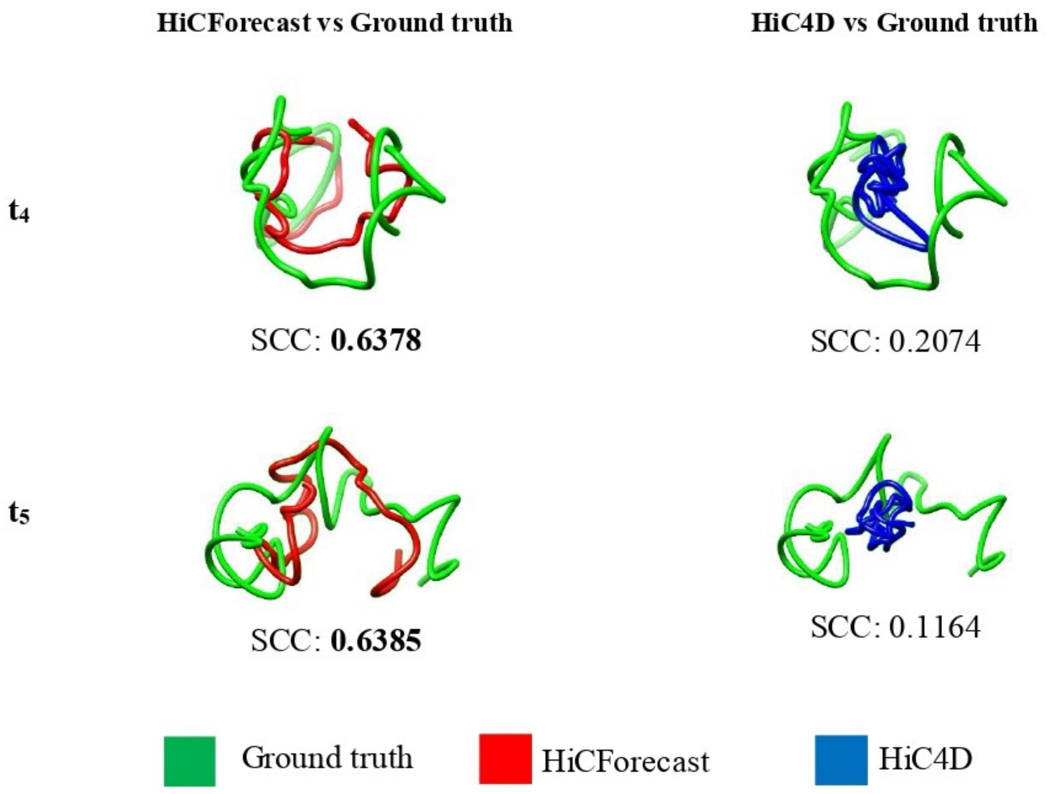

Fig. 17. The comparison of 3D structures of chromosome 2 from Human Embryogenesis (Dataset 3) for genomic region 121Mb to 145Mb. The results show that at timesteps $t_4, and\ t_5$, HiCForecast demonstrates better structural similarity, as per the higher SCC scores.

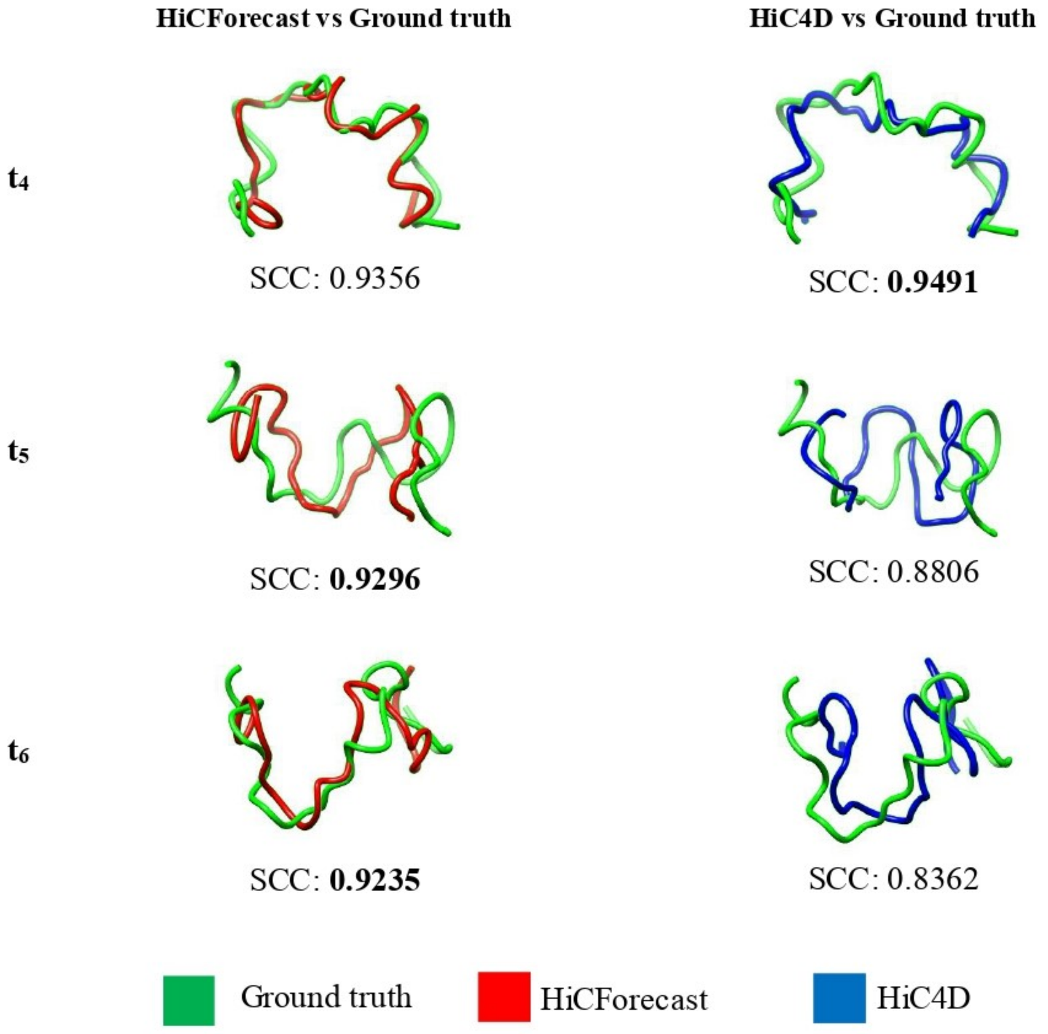

Fig. 18. The comparison of 3D structures of chromosome 6 from Mouse Cell Reprogramming (Dataset 4) for genomic region 121MB to 145MB. The results show that at timesteps $t_5, and\ t_6$, HiCForecast demonstrates better structural similarity, as per the higher SCC scores.

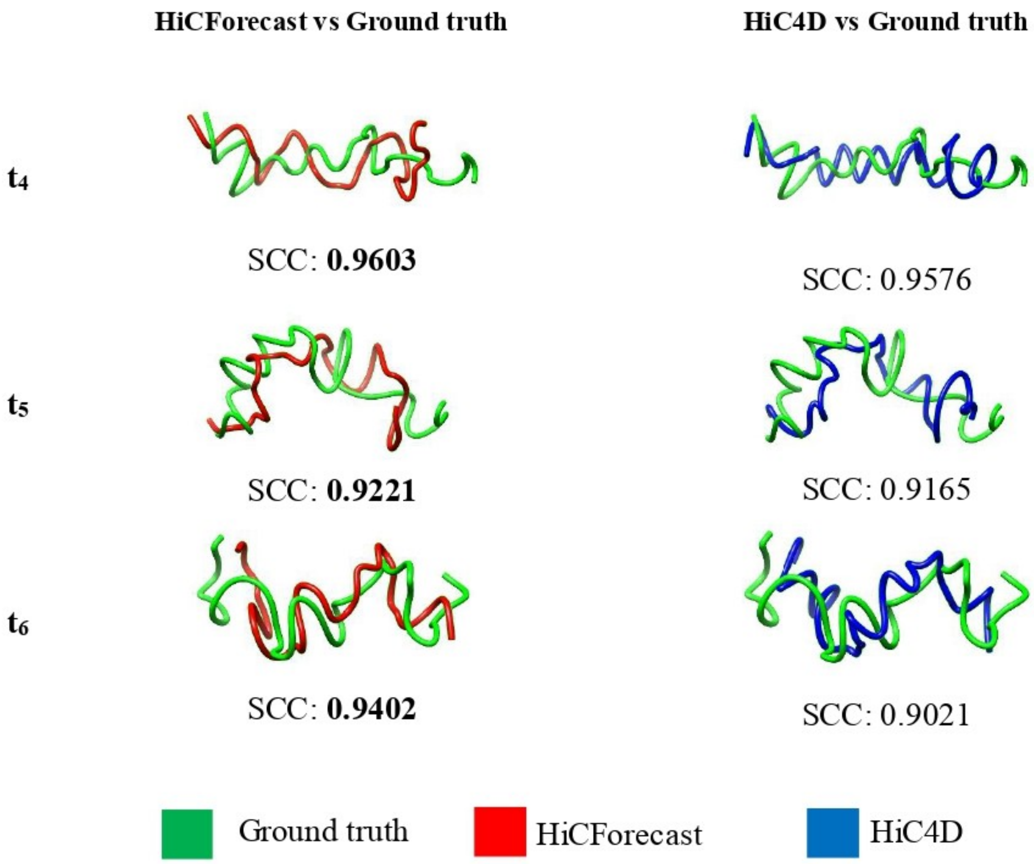

Fig. 19. The comparison of 3D structures of chromosome 6 from Mouse Preimplantation Embryogenesis (Dataset 1) for genomic region 121MB to 145MB. The results show that at timesteps $t_4, t_5, and\ t_6$, HiCForecast demonstrates better structural similarity, as per the higher SCC scores. At timestep t_4, HiC4D demonstrates a slightly higher similarity.

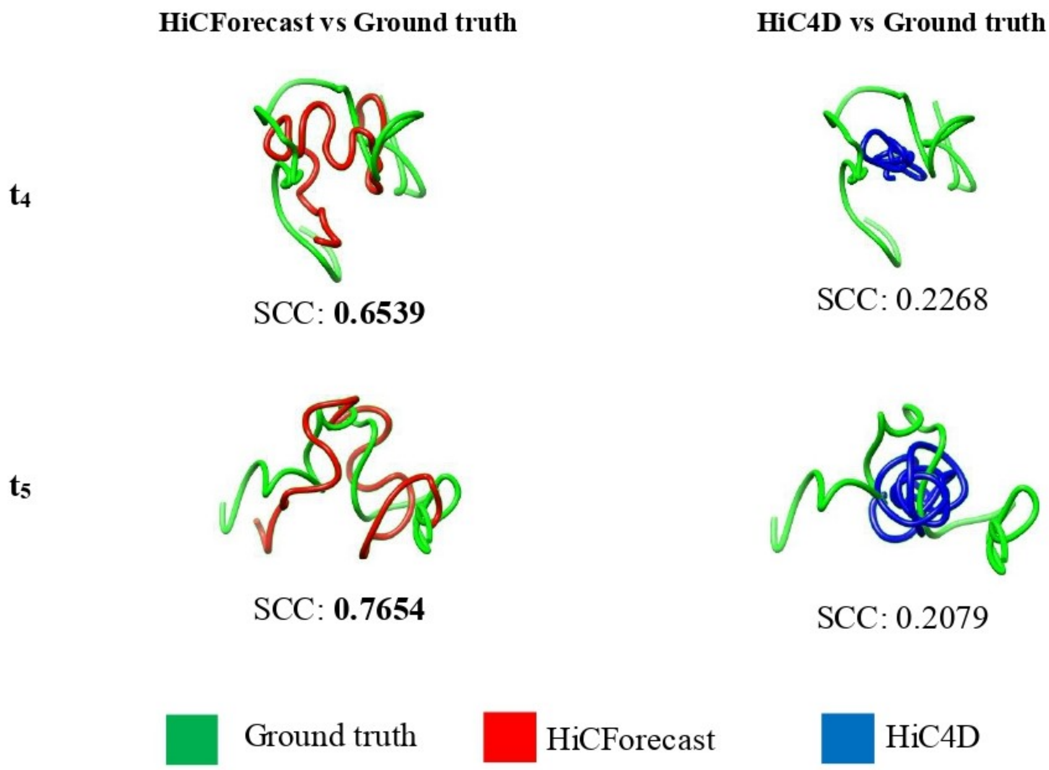

Fig. 20. The comparison of 3D structures of chromosome 6 from Human Embryogenesis (Dataset 3) for genomic region 121MB to 145MB. The results show that at timesteps $t_4, and\ t_5$, HiCForecast demonstrates better structural similarity, as per the higher SCC scores.

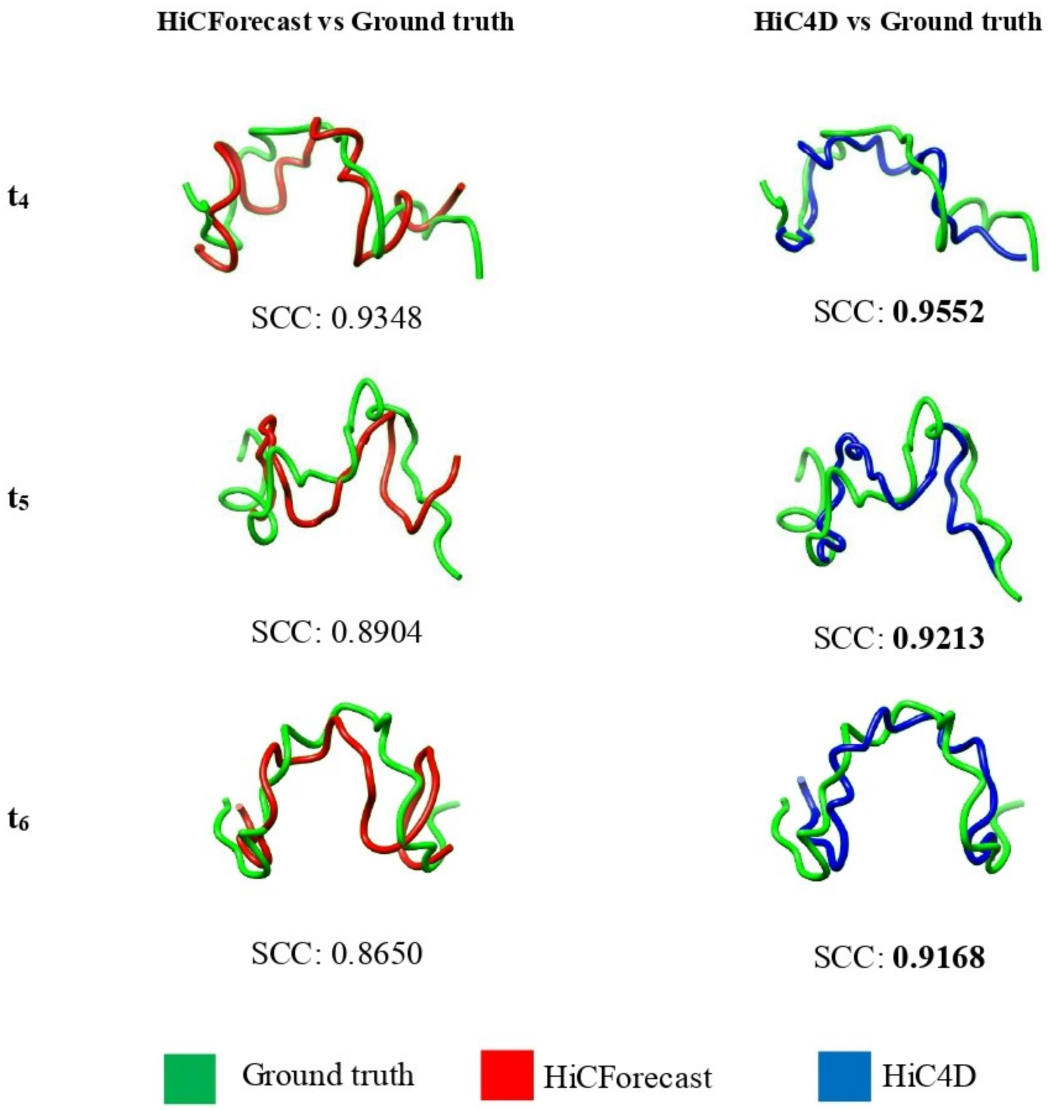

Fig. 21. The comparison of 3D structures of chromosome 2 from Mouse Cell Reprogramming (Dataset 4) for genomic region 121Mb to 145Mb. The results show that HiC4D performs better for all timesteps for this sataset.

References

1. Kellen G Cresswell and Mikhail G Dozmorov. Tadcompare: an r package for differential and temporal analysis of topologically associated domains. *Frontiers in genetics*, 11:158, 2020.
2. Marianna Zazhytska, Albana Kodra, Daisy A Hoagland, Justin Frere, John F Fullard, Hani Shayya, Natalie G McArthur, Rasmus Moeller, Skyler Uhl, Arina D Omer, et al. Non-cell-autonomous disruption of nuclear architecture as a potential cause of covid-19-induced anosmia. *Cell*, 185(6):1052–1064, 2022.
3. Ryohei Nakamura, Yuichi Motai, Masahiko Kumagai, Candice L Wike, Haruyo Nishiyama, Yoichiro Nakatani, Neva C Durand, Kaori Kondo, Takashi Kondo, Tatsuya Tsukahara, et al. Ctcf looping is established during gastrulation in medaka embryos. *Genome Research*, 31(6):968–980, 2021.
4. Longjian Niu, Wei Shen, Zhaoying Shi, Yongjun Tan, Na He, Jing Wan, Jialei Sun, Yuedong Zhang, Yingzhang Huang, Wenjing Wang, et al. Three-dimensional folding dynamics of the xenopus tropicalis genome. *Nature Genetics*, 53(7):1075–1087, 2021.
